# Compartment Model Predicts VEGF Secretion and Investigates the Effects of VEGF Trap in Tumor-Bearing Mice

**DOI:** 10.3389/fonc.2013.00196

**Published:** 2013-07-30

**Authors:** Stacey D. Finley, Manjima Dhar, Aleksander S. Popel

**Affiliations:** ^1^Department of Biomedical Engineering, Johns Hopkins University School of Medicine, Baltimore, MD, USA

**Keywords:** systems biology, mathematical model, computational model, angiogenesis, tumor xenograft model, anti-angiogenic therapy, cancer

## Abstract

Angiogenesis, the formation of new blood vessels from existing vasculature, is important in tumor growth and metastasis. A key regulator of angiogenesis is vascular endothelial growth factor (VEGF), which has been targeted in numerous anti-angiogenic therapies aimed at inhibiting tumor angiogenesis. Systems biology approaches, including computational modeling, are useful for understanding this complex biological process and can aid in the development of novel and effective therapeutics that target the VEGF family of proteins and receptors. We have developed a computational model of VEGF transport and kinetics in the tumor-bearing mouse, which includes three-compartments: normal tissue, blood, and tumor. The model simulates human tumor xenografts and includes human (VEGF_121_ and VEGF_165_) and mouse (VEGF_120_ and VEGF_164_) isoforms. The model incorporates molecular interactions between these VEGF isoforms and receptors (VEGFR1 and VEGFR2), as well as co-receptors (NRP1 and NRP2). We also include important soluble factors: soluble VEGFR1 (sFlt-1) and α-2-macroglobulin. The model accounts for transport via macromolecular transendothelial permeability, lymphatic flow, and plasma clearance. We have fit the model to available *in vivo* experimental data on the plasma concentration of free VEGF Trap and VEGF Trap bound to mouse and human VEGF in order to estimate the rates at which parenchymal cells (myocytes and tumor cells) and endothelial cells secrete VEGF. Interestingly, the predicted tumor VEGF secretion rates are significantly lower (0.007–0.023 molecules/cell/s, depending on the tumor microenvironment) than most reported *in vitro* measurements (0.03–2.65 molecules/cell/s). The optimized model is used to investigate the interstitial and plasma VEGF concentrations and the effect of the VEGF-neutralizing agent, VEGF Trap (aflibercept). This work complements experimental studies performed in mice and provides a framework with which to examine the effects of anti-VEGF agents, aiding in the optimization of such anti-angiogenic therapeutics as well as analysis of clinical data. The model predictions also have implications for biomarker discovery with anti-angiogenic therapies.

## Introduction

Angiogenesis is the formation of new blood capillaries from pre-existing vessels, and is a process involved in physiological function, such as exercise and wound healing, as well as disease conditions, including cancer, peripheral and coronary artery diseases, pre-eclampsia, and age-related macular degeneration (AMD). The vascular endothelial growth factor (VEGF) family is a key promoter of angiogenesis and vascular development. The VEGF family includes five ligands: VEGF-A, VEGF-B, VEGF-C, VEGF-D, and placental growth factor (PlGF). One of the most widely studied members is VEGF-A, commonly referred to as VEGF. Alternative splicing of VEGF produces different isoforms, including VEGF_121_, VEGF_165_, VEGF_189_, and VEGF_206_ in humans. Expressed rodent isoforms are one amino acid shorter than human isoforms; therefore, the subscripted number is one less. Additionally, there are VEGF_xxxb_ isoforms, which have been shown to be endogenous anti-angiogenic species (1, 2). VEGF promotes angiogenesis by binding to and activating its receptors VEGFR1 and VEGFR2, and co-receptors called neuropilins (NRPs). Signal transduction through the receptors promotes many cellular processes, including cell proliferation, migration, and survival (3). VEGFR1 and VEGFR2 are expressed on endothelial cells (ECs), cancer cells, and other cell types, including bone marrow-derived cells and neurons [see (4) for review]. NRPs are expressed on various cell types, including ECs, tumor cells, and muscle fibers (4).

Angiogenesis has been targeted to treat diseases characterized by reduced vascularization (“pro-angiogenic therapy”) (5, 6) or to inhibit the formation of new blood vessels in conditions leading to hypervascularization (“anti-angiogenic therapy”) (7, 8). Of particular importance is anti-angiogenic therapy targeting tumor vascularization. Bevacizumab (9) is a recombinant monoclonal antibody that neutralizes VEGF and is approved by the Food and Drug Administration to treat colorectal cancer, glioblastoma, kidney cancer, and non-small cell lung cancer. Aflibercept (Regeneron) is a soluble decoy receptor approved to treat metastatic colorectal cancer and wet AMD. The drug is also in clinical trials to evaluate its anti-angiogenic effect on various forms of cancers (10). Aflibercept binds to VEGF more tightly than bevacizumab (11) and forms a 1:1 complex with VEGF and PlGF (12). In addition to therapies that target the VEGF ligand, several tyrosine kinase inhibitors (TKIs) have been developed to target phosphorylation of VEGF receptors, as well as other pro-angiogenic receptors including platelet-derived growth factor (PDGF) receptors and fibroblast growth factor (FGF) receptors (13, 14).

Systems biology approaches, including quantitative experimental methods and mathematical modeling, have been applied to study angiogenesis (15–17). Computational models complement experimental studies and can aid in the development and optimization of effective therapeutics (18). Despite extensive basic science and translational research to develop anti-angiogenic therapies, little is known about the drugs’ mechanism of action, how and why tumors become resistant to the treatment, or the patient population that can benefit most from these drugs. Identifying biomarkers that can be used to predict the patients whose tumors will respond favorably to anti-angiogenic treatment is of great interest (19–21). Computational approaches can shed light upon these issues by providing a framework to generate and test hypotheses related to VEGF kinetics and transport in the body (14, 22).

We have previously developed an experiment-based compartment model of VEGF distribution in non-tumor-bearing mice, which estimates the distribution of VEGF in the body (23). Additionally, the model was used to fit kinetic parameters and to predict the rate at which VEGF is secreted by muscle fibers, which is difficult to measure experimentally *in vivo*. In this work, we present an expanded model that includes a tumor compartment and incorporates several new features: EC secretion of VEGF, soluble factors that influence VEGF levels, and a dynamic tumor volume. These new elements lead to a more physiological model and incorporate experimental observations relevant to VEGF kinetics and transport in the whole body, which can be compared to experimental data. Thus, this work represents a significant expansion to our previous models (23–26). We first re-calibrate the two-compartment model (no tumor is present) using *in vivo* experimental data and estimate the rates at which VEGF is secreted by muscle fibers and ECs, as well as the clearance rates of unbound and complexed VEGF Trap, and the binding affinity of VEGF trap. We then fit the three-compartment model to available *in vivo* experimental data in order to estimate the rate of VEGF secretion by muscle fibers, ECs, and tumor cells. We demonstrate how the model can be applied to investigate the effect of neutralizing VEGF using VEGF Trap. These results contribute to our understanding of the efficacy of VEGF Trap in specific tumor types. We also estimate the concentrations of VEGF in different compartments, which can be validated experimentally.

## Results

### Re-calibration of two-compartment model captures dynamics of bound and complexed VEGF Trap

The previous two-compartment model simulating non-tumor-bearing mice (23) did not include EC secretion of VEGF or soluble factors. Therefore, we first refit the expanded two-compartment model that includes these additional features in order to match *in vivo* experimental data (12). The fitting optimized the values of five parameters: VEGF secretion rate of muscle fibers $\left(q_{\text{VEGF}}^{\text{muscle}}\right)$, VEGF secretion rate of ECs $\left(q_{\text{VEGF}}^{\text{EC}}\right)$, clearance rate of VEGF Trap (*c*_A_), clearance rate of the VEGF/VEGF Trap complex (*c*_VA_), and dissociation constant of VEGF and VEGF Trap (*K*_d_). As described in the methods, although the experimental protocol used by Rudge and coworkers utilizes subcutaneous administration of VEGF Trap, we simulate intravenous administration and assume 100% of the reported dose is administered. The fitting procedure allows us to estimate the values of the free parameters using *in vivo* experimental data.

**Figure 1:**
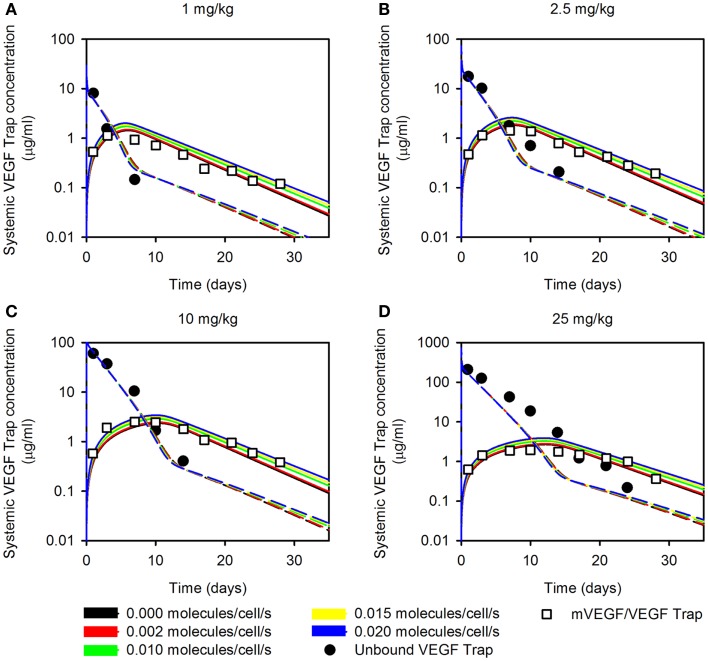
**Effect of varying muscle secretion in two-compartment model**. The estimated plasma levels of free VEGF Trap (dashed lines) and mouse VEGF bound to VEGF Trap (solid lines) after a single intravenous injection of VEGF Trap at **(A)**, 0.5 mg/kg; **(B)**, 2.5 mg/kg; **(C)**, 10 mg/kg; and **(D)**, 25 mg/kg. The rate of VEGF secretion by muscle fibers is varied from 0 to 0.02 molecules/cell/s. Model results are compared to experimental measurements for free VEGF Trap (black circles) and mVEGF bound to VEGF Trap (white squares).

The optimized parameter values are shown in Table 1, and all raw data from the optimization is given in File 1 in Supplementary Material. The optimized value of *K*_d_ is comparable to the reported *in vitro* measurement of 0.6 pM (11), providing confidence in the fitting procedure. The optimization predicts the muscle fibers secrete very little VEGF (0.002 molecules/cell/s), and the standard deviation of the optimized values is high. This suggests that the model is not sensitive to the value of $\left(q_{\text{VEGF}}^{\text{muscle}}\right)$. To investigate this possibility, we varied muscle secretion from 0 to 0.02 molecules/cell/s and used the model to estimate the concentrations of unbound VEGF Trap and the mouse VEGF (mVEGF)/VEGF Trap complex. This sensitivity study revealed that increasing $\left(q_{\text{VEGF}}^{\text{muscle}}\right)$ up to one order of magnitude does not significantly change the fit, as shown in Figure 1. These results indicate that there may not be sufficient data to determine VEGF secretion from muscle fibers. Specifically, it is difficult to separate the contribution of VEGF from muscle fibers, compared to ECs. This result is not specific to the data used here, but more generally that plasma measurements cannot be used to determine endogenous VEGF production from multiple sources.

**Table 1:** **Estimated model parameters from optimization of two-compartment model**.

Parameter	Units	Optimal value	Standard deviation
Normal secretion	Molecules/cell/s	0.002	0.003
EC secretion	Molecules/cell/s	0.057	0.004
Tumor secretion	Molecules/cell/s	N/A	N/A
Clearance of free VEGF Trap	s^−1^	1.3 × 10^−5^	2 × 10^−7^
Clearance of bound VEGF Trap	s^−1^	2.5 × 10^−6^	2 × 10^−7^
*K*_d_ of VEGF Trap	pM	0.29	0.011

### Sensitivity analysis reveals model parameters that influence VEGF concentrations

In the three-compartment model, the values of several parameters are based on characterization of the human VEGF (hVEGF) system due to a lack of quantitative experimental measurements in mice. We previously investigated sensitivity to individual parameters, including vascular permeability, lymphatic drainage, and properties of the anti-VEGF agent (25). In that work, parameters were varied one by one. Here, we perform a modular sensitivity analysis, where we investigate how variability in three sets of parameters (model inputs) influence mouse and hVEGF concentrations and sVEGFR1 levels in normal tissue, blood, and tumor (model outputs). Specifically, we investigated the effect of VEGF receptor expression, transport parameters, and kinetic parameters using the extended Fourier Amplitude Sensitivity Test (eFAST), as described in the Section “Materials and Methods.” Two indices provide an estimate of the sensitivity of the model output to model parameters. The first FAST index quantifies the variance of a model output with respect to the variance of each input. The total FAST index quantifies the variance of a model output with respect to the variances of each input and covariances between all combinations of inputs. If total FAST indices are larger than the first FAST indices, it means that the parameter is more important in combination with other parameters rather than individually.

**Figure 2:**
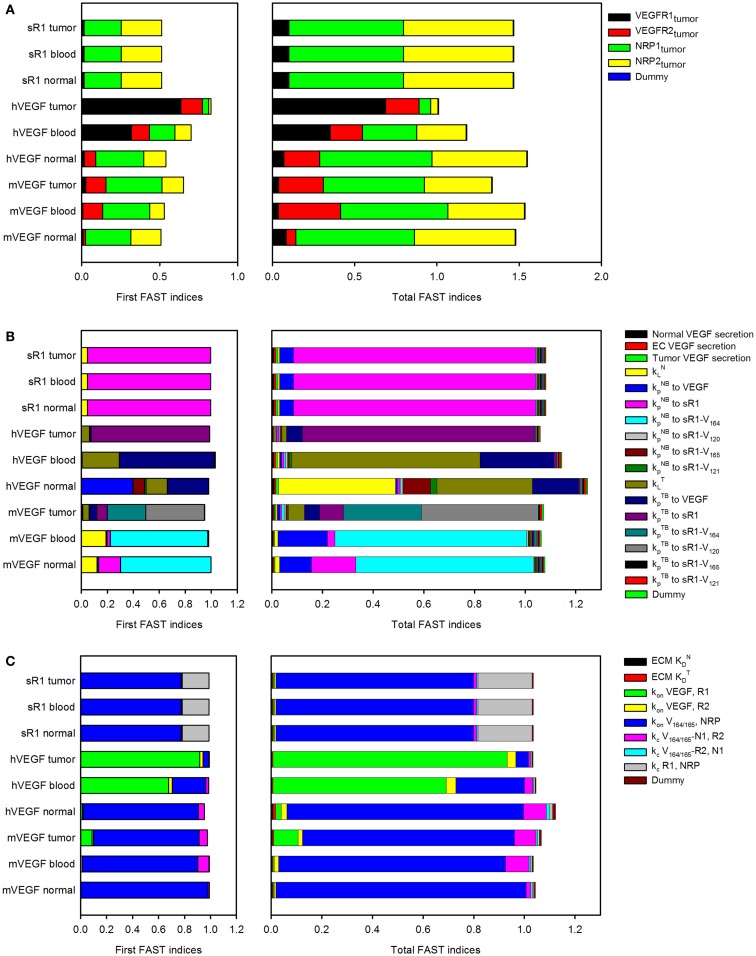
**Sensitivity indices of model parameters**. The extended Fourier Amplitude Sensitivity Test (eFAST) was used to estimate the variance in the model output with respect with variance in individual model inputs (first FAST indices) and covariances in combinations of model inputs (total FAST indices). A modular approach was used to investigate the sensitivity to **(A)**, tumor receptor expression; **(B)**, transport parameters; and **(C)**, kinetic parameters.

The FAST indices for each set of model inputs are shown in Figure 2. When investigating the effect of tumor cell receptor expression, VEGF and sVEGFR1 concentrations are sensitive to the density of NRP co-receptors. Additionally, the level of VEGFR1 is an important determinant of hVEGF concentration in the tumor. In the transport module, the rate of lymphatic flow from normal or tumor tissue in concert with other transport parameters is estimated to influence hVEGF levels in plasma and normal tissue. Soluble VEGFR1 concentrations, as well as mVEGF levels in plasma and normal tissue, are particularly sensitive to the permeability of the normal tissue to VEGF and VEGF/sVEGFR1 complexes. Individual parameters investigated in the kinetic module are predicted to influence VEGF and sVEGFR1 concentrations, rather than in combination with other kinetic parameters. VEGF and sVEGFR1 levels are particularly sensitive to VEGF_164_ and VEGF_165_ binding to NRP co-receptors and VEGF binding to VEGFR1. These results aid in our understanding of how uncertainty in the values of particular parameters influence the model output. Additionally, the sensitivity analysis provides quantitative data to support obtaining additional experimental measurements of specific parameters that significantly influence model outputs.

### The rate of VEGF secretion by human tumor cells is dependent on the tumor microenvironment

Tumor cells are a source of VEGF; however, there is a lack of *in vivo* data for VEGF secretion rates. Therefore, we have used *in vivo* experimental data on the plasma concentration of free VEGF Trap and VEGF Trap bound to mouse and hVEGF to determine VEGF secretion rates in mice bearing human tumor xenografts. Here, we use the clearance rates of unbound and complexed VEGF Trap predicted in the two-compartment model and experimentally determined VEGF binding affinity. However, the VEGF secretion rates ($q_{\text{VEGF}}^{\text{muscle}}$, $q_{\text{VEGF}}^{\text{EC}}$, and tumor VEGF secretion, $q_{\text{VEGF}}^{\text{tumor}}$) were optimized to fit experimental data. We optimize the VEGF secretion rates since there is large variability in the predicted rate of muscle secretion obtained using the two-compartment model.

**Figure 3:**
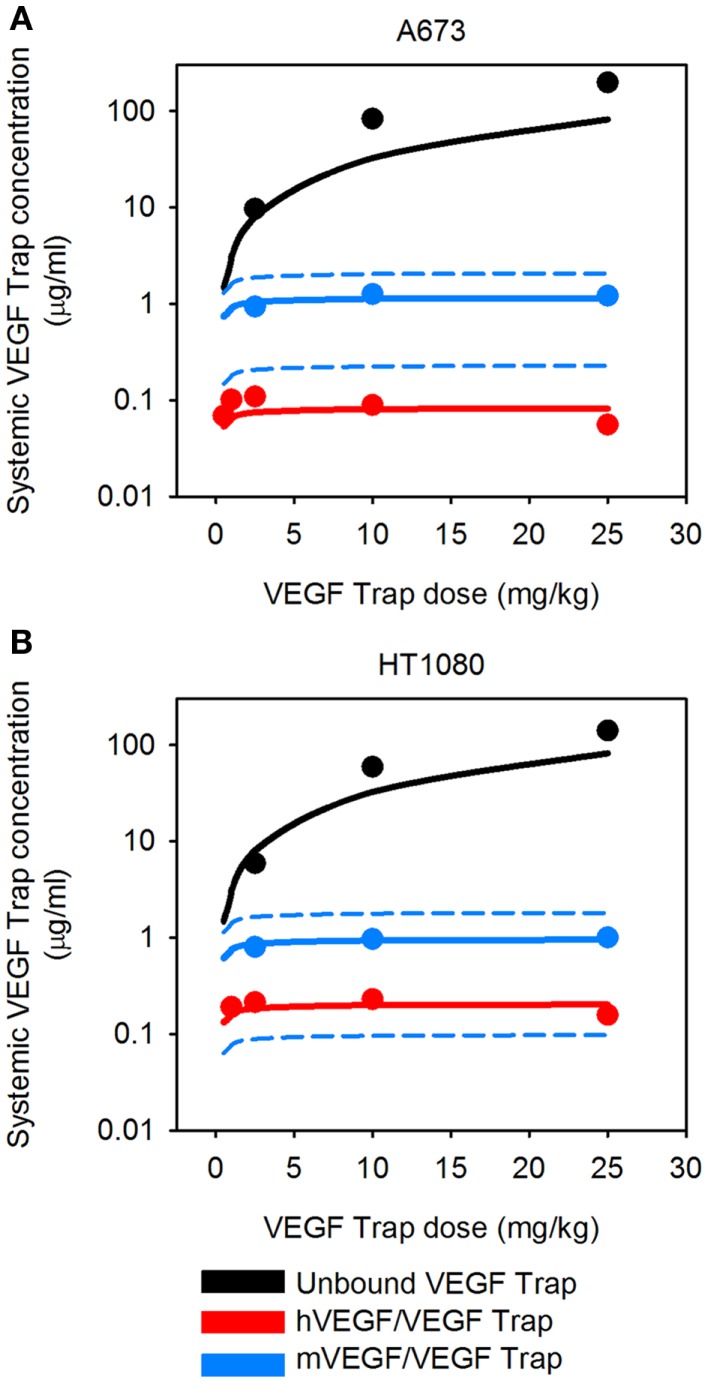
**Predicted systemic VEGF Trap levels**. The model predicts the plasma levels of free VEGF Trap (black lines), mouse VEGF bound to VEGF Trap (blue lines), and human VEGF bound to VEGF Trap (red lines). VEGF Trap was administered twice per week for 2 weeks at doses of 0.5, 1, 2.5, 10, and 25 mg/kg. The simulated results are shown for the optimized model where the secretion rates of VEGF by myocytes, EC, and tumor cells were fit to experimental data (circles). We use the mean (solid lines) and 1 SD (dashed lines) of the fitted secretion rates. **(A)** A673 tumor; and **(B)**, HT1080 tumor. Results for fast-growing tumor are in Figure A1 in Appendix.

**Figure 4:**
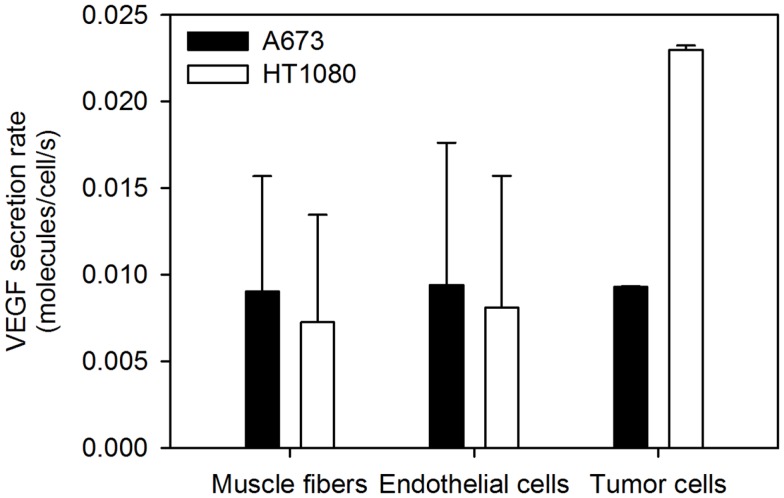
**Optimized VEGF secretion rates**. The model parameters were optimized to fit experimental data, and the values of normal, EC, and tumor VEGF secretion rates were determined. The mean optimal secretion rates and standard deviation of 20 optimization runs are shown. Results for fast-growing tumors are in Figure A2 in Appendix.

The VEGF secretion rates were predicted using the optimization algorithm, assuming the tumors follow either the average (baseline) or fast tumor growth profiles. We use data from Rudge et al. (12), where tumors were allowed to grow to ∼100 mm^3^, and then the tumor-bearing mice were injected with VEGF Trap (“anti-VEGF”) twice weekly for 2 weeks. Various dosages of VEGF Trap were used, and the concentrations of free VEGF Trap and the mVEGF/VEGF Trap complex and hVEGF/VEGF Trap complex in the blood were measured. These measurements can be directly compared to model estimates where the anti-VEGF agent is administered intravenously. The optimized model provides a good fit to the experimental data, as shown in Figure 3. The average and standard deviation of the predicted VEGF secretion rates from the optimization runs are in Figure 4 and Table 2, and File 1 in Supplementary Material contains the raw data.

**Table 2:** **Estimated VEGF secretion rates from optimization of three-compartment model**.

Tumor	Baseline tumor growth profile*			Fast growth profile		
	Normal	EC	Tumor	Normal	EC	Tumor
A673	0.011 ± 0.007	0.009 ± 0.008	0.009 ± 5 × 10^−5^	0.009 ± 0.006	0.009 ± 0.008	0.007 ± 4 × 10^−5^
HT1080	0.007 ± 0.006	0.008 ± 0.008	0.023 ± 3 × 10^−4^	0.007 ± 0.006	0.008 ± 0.008	0.017 ± 3 × 10^−5^

*Secretion rate is given in molecules/cell/s. We report the mean ± SD of the 20 optimization runs.

### Circulating levels of VEGF Trap and human VEGF/VEGF Trap complex and maximum concentration of total VEGF Trap vary with dose

**Figure 5:**
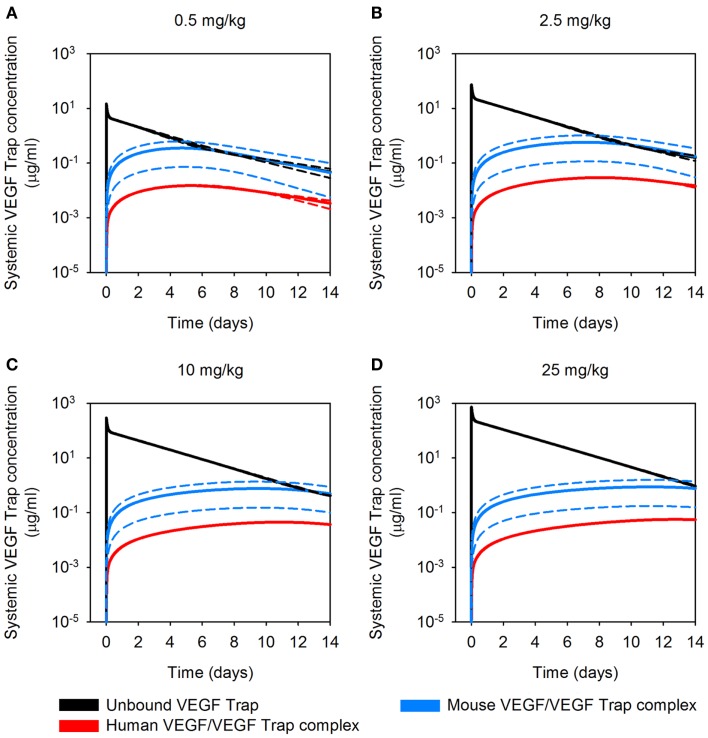
**Predicted concentration profiles of systemic VEGF Trap**. The optimized model was applied to predict the time course of free VEGF Trap (black), mouse VEGF bound to VEGF Trap (blue), and human VEGF bound to VEGF Trap (red) in the mouse plasma after a single intravenous injection of VEGF Trap at **(A)**, 0.5 mg/kg; **(B)**, 2.5 mg/kg; **(C)**, 10 mg/kg; and **(D)**, 25 mg/kg in the A673 rhabdomyosarcoma human tumor xenograft. We use the mean (solid lines) and standard deviation (dashed lines) of the fitted secretion rates.

To our knowledge, the dynamic levels of free and complexed VEGF Trap in tumor-bearing mice have not been reported. These data are useful in elucidating the mechanism of action of VEGF Trap and to determine if the dosage is sufficient to neutralize VEGF secreted by the tumor. Therefore, we used the optimized model for A673 rhabdomyosarcoma human xenograft to predict the concentration profiles for free VEGF Trap and VEGF Trap bound to hVEGF (Figure 5). The level of VEGF Trap bound to hVEGF is more than an order of magnitude lower than the concentration of mVEGF complexed with VEGF Trap. This result is consistent with the finding that normal production of VEGF eclipses the production from tumors, as described by Rudge and co-authors (12). Additionally, the level of free VEGF Trap remains well above the level of the hVEGF/VEGF trap complex for up to 14 days. This indicates effective dosing, as the VEGF-neutralizing agent is able to neutralize all VEGF secreted by the tumor. The HT1080 fibrosarcoma tumor response is similar (data not shown).

### VEGF Trap is predicted to deplete unbound VEGF in the body

**Figure 6:**
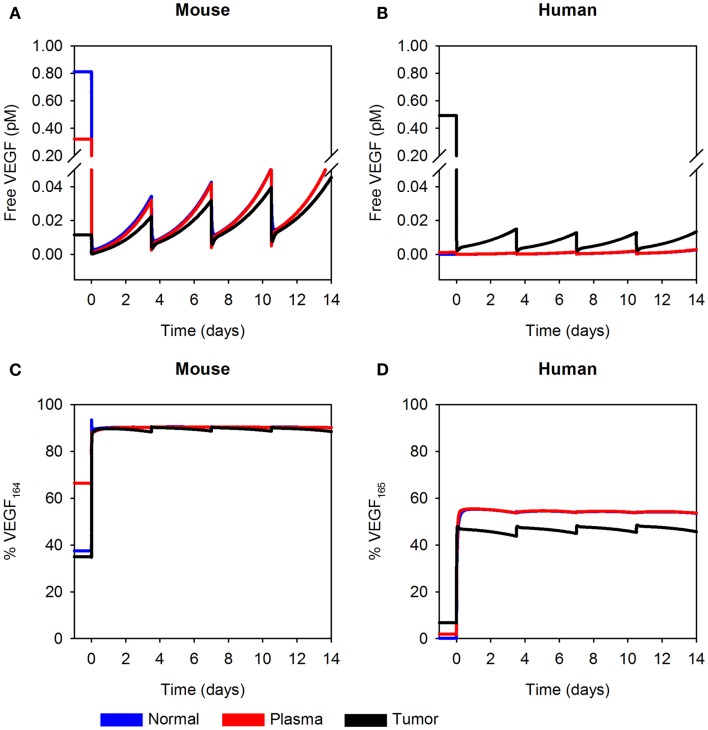
**Predicted concentration profiles of unbound VEGF**. The optimized model was applied to predict the time course of free VEGF and the fraction of free VEGF in the form of VEGF_164_ or VEGF_165_ in normal tissue (blue), plasma (red), and tumor (black) twice-weekly injection of VEGF Trap at 2.5 mg/kg. **(A)** Free mVEGF; **(B)**, free hVEGF; **(C)**, percentage of VEGF_164_; and **(D)**, percentage of VEGF_165_ in the A673 rhabdomyosarcoma human tumor xenograft. We use the mean of the fitted secretion rates.

The optimized model of a tumor-bearing mouse provides a framework with which to study the concentration of unbound VEGF before and after administration of VEGF Trap. As expected, endogenous levels of unbound VEGF are highest in the normal tissue and plasma, and the concentration of hVEGF is highest in the tumor, based on the source of mouse and hVEGF. Before any injection, mVEGF concentration is estimated to range from 0.17 to 1.47 pM in mice with A673 tumors, based on 1 SD above and below the average predicted VEGF secretion rates (Table 3). Unbound hVEGF in the tumor is estimated to be ∼0.5 pM. We also present free VEGF concentration during twice-weekly injections of VEGF Trap at 2.5 mg/kg (Figures 6A,B). The model estimates that free VEGF in the body is first depleted before increasing slightly before the next injection. Thus, the model can be used to understand the effect of anti-VEGF agents on systemic and tissue levels of VEGF.

**Table 3:** **Estimated concentrations of free VEGF before VEGF Trap injection**.

Tumor	Range of free VEGF (pM)*					
	Mouse			Human		
	Normal	Plasma	Tumor	Normal	Plasma	Tumor
A673	0.17–1.47	0.04–0.61	0.002–0.02	5.03 × 10^−5^–5.30 × 10^−5^	1.18 × 10^−3^–1.20 × 10^−3^	0.49–0.50
HT1080	0.07–1.27	0.02–0.54	0.001–0.02	1.26 × 10^−4^–1.34 × 10^−4^	2.95 × 10^−3^–3.05 × 10^−3^	1.23–1.26

*Calculated using (mean ± SD).

In addition to using the model to estimate the concentration of unbound VEGF, we have also determined the percentage of free VEGF in the form of VEGF_164_ or VEGF_165_. The isoform secretion ratio for VEGF_164_:VEGF_120_ in muscle is 92:8 and 90:10 in EC, and the secretion ratio for VEGF_165_:VEGF_121_ in tumor cells is 50:50, as described in the Section “Materials and Methods.” These ratios determine the fraction of VEGF_164_ or VEGF_165_ in the compartments; and, the fractions at which the isoforms are present change with time and drug dose. Here, we consider a dosage of 2.5 mg/kg. After the first anti-VEGF injection, the percentage of free mVEGF in the form of VEGF_164_ is ∼90% in all compartments (Figure 6C, left). The percentage of hVEGF in the form of VEGF_165_ in tumor is slightly lower than the percentage of VEGF_165_ in normal tissue and plasma (44–49%, as compared to 55%; Figure 6D). These types of model predictions can aid in biomarker identification, as the concentration of specific VEGF isoforms may predict tumors that will respond to anti-VEGF treatment or other anti-angiogenic therapies.

We also apply the model to investigate the total levels of circulating VEGF in plasma. The soluble factors sVEGFR1 and α-2-macroglobulin (α2M) bind to VEGF and contribute to circulating levels of VEGF. Thus, total circulating VEGF is comprised of free VEGF, VEGF bound to sVEGFR1, and α2M-bound VEGF (both the native and active forms). VEGF bound to the VEGF Trap drug is also included. We again allow the tumors to reach a volume of 100 mm^3^ before simulating twice-weekly injections of VEGF Trap at varying doses. Before the first injection, the relative amounts of free, sVEGFR1-bound, and α2M-bound circulating VEGF are 80, 4, and 16%, respectively. One day after the first injection of VEGF Trap, the composition of the circulating VEGF changes, depending on the drug dose (data not shown). If we consider a drug dose of 2.5 mg/kg, the relative amounts of free, sVEGFR1-bound, α2M-bound, and VEGF Trap-bound VEGF are 0.6, 0.03, 5, and 94%, respectively. Thus, the VEGF Trap displaces the soluble factors bound to VEGF.

## Discussion

We have developed a compartment model of VEGF distribution in tumor-bearing mouse. The model incorporates tumor-specific properties, including the rate of tumor growth and VEGF secretion. We have used *in vivo* experimental data for the levels of free and bound VEGF Trap in mice bearing human tumor xenografts in order to predict the endogenous rate of VEGF secretion by myocytes and ECs and compared them to the predicted secretion rates in normal mice. We also predicted the rate at which cells from different human tumor xenografts secrete VEGF. To our knowledge, VEGF secretion rates can only be obtained from *in vitro* experiments and cannot be directly measured *in vivo*; however, VEGF concentrations that depend on the secretion rates can be measured experimentally, although such interstitial measurements are presently not available. Therefore, this work provides new insight into VEGF levels in a pre-clinical *in vivo* model of cancer. In addition, using the optimized model for tumor-bearing mice, we have estimated the concentration of VEGF in the mouse following administration of VEGF Trap, as well as the distribution of VEGF in mice and circulating levels of VEGF Trap and the VEGF/VEGF Trap complex. These results show that the concentration of free VEGF in the tumor depends on the tumor-specific properties such as the rate of tumor growth and the amount of VEGF secreted by tumor cells. Lastly, we used the predicted level of VEGF Trap and hVEGF/VEGF Trap complex to compare various dosages. The model predicted that all hVEGF originating from the tumor is neutralized at higher doses of the drug. This demonstrates an important application of the model: to incorporate tumor-specific properties and investigate the efficacy of different drug doses.

We used the two-compartment model to estimate VEGF secretion rates, clearance of free and bound VEGF Trap, and the binding affinity of VEGF Trap for normal mice. The value of binding affinity of VEGF Trap estimated by the model is comparable to the experimentally measured value (11). Additionally, the estimated EC secretion is comparable to the experimentally determined value of 0.028 molecules/cell/s (27). However, the predicted rate at which muscle cells secrete VEGF is very low, and varying this parameter over one order of magnitude does not significantly change the fit. In contrast, EC secretion can be specified and changing this parameter drastically influences the fit to experimental data (results not shown). These results may indicate that the rate of VEGF secretion from muscle and ECs cannot be simultaneously estimated using the available experimental data. That is, measurements of free and bound VEGF Trap in plasma do not allow us to distinguish how muscle and ECs contribute to VEGF levels. Additional experimental measurements such as interstitial levels of VEGF in skeletal muscle are needed in order to predict VEGF secretion by muscle fibers with confidence. Currently, interstitial VEGF concentrations are only available in human tissue (28–33); however, similar studies in mice are of great interest.

We found that fitted parameters from normal mice were not sufficient to match the levels of unbound and complexed VEGF Trap in the model of tumor-bearing mice. We first attempted to use the fitted parameters from the two-compartment model in the model of tumor-bearing mice and use *in vivo* experimental data to fit the rate of VEGF secretion from tumor cells. However, the model overestimated the amount of VEGF Trap complexed with mVEGF (results not shown). We are able to more closely fit the experimental data for the tumor-bearing mice by optimizing the three-compartment model independent of the optimized model for normal mice. This indicates that endogenous VEGF secretion may be different in normal and tumor-bearing mice (Tables 1 vs 2). Experimental studies are needed to validate these results; however, evidence shows that VEGF secretion is reduced following administration of VEGF Trap (34) or other anti-angiogenic therapies (35–37).

The three-compartment model predicted that the *in vivo* tumor VEGF secretion rates needed to fit experimental data are lower than data obtained from *in vitro* measurements. *In vitro* experimental measurements of the VEGF secretion rate vary widely: 0.03–2.65 molecules/cell/s (38– 41). We predicted that human tumors secrete VEGF at rates range ranging from 0.007 to 0.023 molecules/cell/s. Interestingly, there is little variability in the predicted tumor secretion rate, as indicated by the small standard deviation (∼10^−5^ molecules/cell/s). Having experimental measurements of the plasma concentration of VEGF Trap bound to hVEGF (i.e., VEGF originating from the tumor) enables us to predict the rate at which the tumor secretes VEGF *in vivo*. In this way, xenograft models are preferable to syngeneic tumor models, in which VEGF derived from tumor and other tissues are indistinguishable. Similarly, plasma measurements in human patients would not be sufficient to specify tumor VEGF. Thus, xenograft models provide unique insight into the effects of anti-angiogenic therapies and are relevant to human studies.

Tumor VEGF secretion is predicted to depend on the tumor microenvironment. HT1080 tumors are predicted to secrete ∼2-fold more VEGF than A673 tumors. Additionally, average- and fast-growing tumors are predicted to secrete different amounts of VEGF, where VEGF secretion in fast-growing tumors is slightly lower than that of tumors that grow at an average rate. To our knowledge, experimental data for VEGF secretion rates is limited to *in vitro* measurements. Therefore, the ability to use the model to determine the VEGF secretion from *in vivo* data and track and quantify normal and tumor VEGF are important features of the model.

Using the optimized model, it is possible to estimate VEGF concentrations in the mouse before and after VEGF Trap administration. In the model, we allowed the tumor to grow for 2 weeks before the VEGF Trap injection. Just before the injection, the estimated plasma VEGF levels are within the range of experimental measurements in mouse of 0.3–1.4 pM (42, 43). The model indicates that plasma VEGF depends on properties of the tumor, such as volume, a result that is validated by experimental evidence (44). Using the model, free VEGF in muscle interstitium is predicted to range from 0.2 to 1.5 pM. To our knowledge, interstitial VEGF in normal tissues has only been quantified in human samples. Interstitial muscle VEGF in humans ranges from 0.3 to 3 pM (28– 33, 45). It is not clear how this concentration range varies across species. However, since the range of plasma VEGF measurements is similar between mice and humans, where human plasma VEGF is measured to be 0.4–3 pM (46), it is possible that interstitial VEGF is also comparable in mice and humans. Thus, our model results and predictions provide a framework to compare VEGF distribution in different species and can be experimentally validated. Additionally, we are able to predict the concentration of specific VEGF isoforms (i.e., the percentage of free VEGF in the form of VEGF_164_ or VEGF_165_, as compared to the shorter isoforms VEGF_120_ or VEGF_121_). These results may be useful in identifying predictive biomarkers for anti-VEGF treatment, where the level of VEGF_121_ is being evaluated as a biomarker (47, 48). We also applied the model to estimate the relative contribution of sVEGFR1-bound and α2M-bound VEGF to total circulating VEGF. The soluble factors compete with anti-VEGF agents; therefore, it is of interest to investigate the effect of sVEGFR1 on the response to anti-VEGF treatment. In this way, the model complements studies evaluating sVEGFR1 as a potential biomarker to predict resistance to anti-VEGF treatment (49).

We can also compare the estimated levels of plasma VEGF generated by the model following administration of VEGF Trap with experimental studies. *In vivo* studies of mice with breast tumor xenografts indicate the plasma VEGF is reduced following VEGF Trap treatment, particularly at the higher doses (34). Additionally, Hoff and coworkers report that VEGF Trap is able to bind all free VEGF 11 days after treatment in an experimental model of rat glioma (50). These studies support the computational model predictions. However, we are not aware of animal studies that provide the time course of VEGF and VEGF/VEGF Trap concentration, which is an important contribution of the model and can complement pre-clinical studies that investigate the efficacy of VEGF Trap.

We show that interstitial tumor VEGF levels depend on specific properties of the tumor. To our knowledge, there are no experimental measurements for interstitial tumor VEGF concentrations. However, a sampling of available experimental measurements of total VEGF in tumor tissue (free and bound VEGF, both intracellular and extracellular) reveals a wide range of values, depending on tumor type and size. File 1 in Supplementary Material shows a compilation of measurements of tumor VEGF for various tumor types. Experimental studies to measure free VEGF in tumor tissue in mouse models would provide much needed quantitative data to test and validate the model predictions presented here.

### Model limitations

We consider the model presented here to be a minimal model that accurately reproduces experimental data, both qualitatively and quantitatively. The model includes several assumptions, which may be addressed as quantitative data become available. For example, we assume the normal tissue is skeletal muscle, although other tissues and organs secrete and contain VEGF (51), but are not as well-characterized as muscle. We include two major VEGF isoforms (VEGF_120_/VEGF_121_ and VEGF_164_/VEGF_165_); however, other isoforms such as VEGF_188_/VEGF_189_ (52) and VEGF_xxxb_ (53, 54) also influence angiogenesis and may impact anti-VEGF therapies. Recent studies also show that other VEGF ligands and receptors contribute to angiogenesis (55–57), and the model can be expanded in the future to include these molecular species. Additionally, although platelets contain large amounts of VEGF and contribute to angiogenesis (58), we have not included them in the model as the rate and conditions under which they secrete or unload VEGF are unknown. We assume that as the tumor grows, the relative proportions of interstitial space, vascular volume, and tumor cells remain constant. However, experimental studies indicate that these proportions should change as the tumor grows (59). Finally, we have not included the effects of anti-VEGF treatment on tumor volume or vascular permeability. Pre-clinical studies show tumor growth inhibition and even regression of the tumor following anti-angiogenic therapy that targets VEGF. We have performed preliminary studies where the tumor volume is constant after 1 week of anti-VEGF treatment since experimental studies indicate that tumor growth is halted during 2 weeks of twice-weekly VEGF Trap injections (34). We found that the predicted tumor secretion rate is slightly larger when accounting for tumor growth stagnation. This is because the tumor is smaller and consists of fewer cells. Therefore, the amount of VEGF that must be secreted on a per cell basis in order to obtain a certain level of VEGF or VEGF/VEGF Trap complex is higher. Tumor permeability may decrease with anti-angiogenic therapy, as the tumor normalizes neovasculature and it begins to resemble normal vessels; however, we have not included that effect in the current model. In a human model of VEGF transport and kinetics, we considered “low” and “high” vascular permeability between the tumor and blood (22). Interestingly, the model predicts that tumor VEGF can increase above the pre-treatment level depending on properties of the tumor microenvironment, even when tumor permeability is high. Future computational studies may investigate the effect of anti-VEGF treatment on tumor volume and vascular permeability in greater detail.

### Conclusion

The compartment model presented here provides a framework to investigate the action of VEGF-targeting agents for particular types of tumors. The physiologically based and experimentally validated model, based on currently available animal data, predicted the dynamic concentrations of molecular species and other biological parameters that are difficult to quantify experimentally. Thus, the model complements pre-clinical experiments, can aid in the development of agents that target VEGF and inhibit angiogenesis, and may be useful in evaluating biomarkers of anti-angiogenic therapies. The model can be extended to human patients; this is particularly important since in 2012 aflibercept has been approved to treat metastatic colorectal cancer in humans (60).

## Materials and Methods

### Computational model

**Figure 7:**
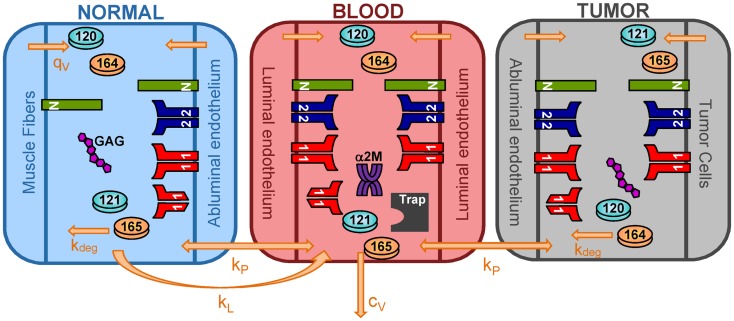
**Three-compartment model of VEGF**. The model is comprised of three-compartments: normal tissue, blood, and tumor. VEGF_120_ and VEGF_164_ are secreted by myocytes in the normal tissue and by EC in all compartments. Tumor cells secrete the human isoforms VEGF_121_ and VEGF_165_. VEGF receptors (VEGFR1 and VEGFR2) and co-receptors (NRPs) are localized on parenchymal and endothelial cells. Soluble VEGFR1 and glycosaminoglycan (GAG) chains are present in the interstitial space. Alpha-2-macroglobulin (α2M) is present in the blood. Molecular species are transported between compartments via microvascular permeability (*k*_p_) and lymphatic drainage (*k*_L_). All isoforms of unbound VEGF in the tissue compartments are subject to proteolytic degradation (*k*_deg_) and are removed from the blood via plasma clearance (*c*_V_).

**Figure 8:**
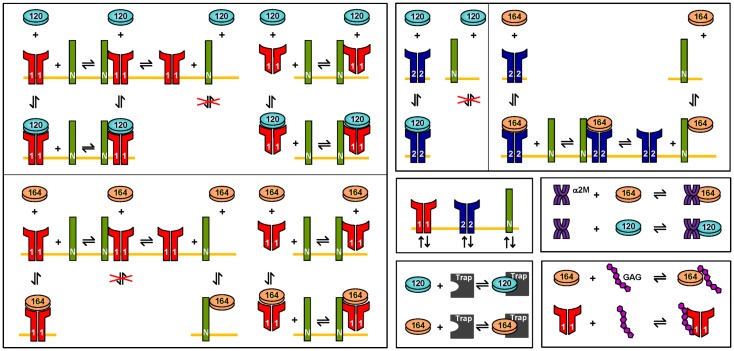
**Molecular interactions**. The interactions of VEGF_120_ and VEGF_164_ are illustrated. VEGF_121_ is involved in the same binding reactions as VEGF_120_. Similarly, the interactions for VEGF_165_ are the same as VEGF_164_. Differences in the interactions of VEGF_120/121_, as compared to VEGF_164/165_ are due to differential exon splicing.

We have expanded the two-compartment model of VEGF distribution in the mouse (23) to include tumor tissue (“tumor compartment”). The model is illustrated in Figure 7. Geometric and kinetic parameters for the normal and blood compartments have been fully detailed in (23). By simulating a human tumor xenograft (tissue that grows from human cancer cells that have been injected into the mouse), we also incorporate hVEGF isoforms and cross-species reactions between ligands and receptors. Specifically, we include VEGF_121_ and VEGF_165_, which are secreted by tumor cells. The human isoforms can bind to human receptors present on tumor cells, as well as mouse receptors on endothelial surfaces in the body (normal and tumor EC) and muscle fibers in the normal compartment. Additionally, the mouse isoforms bind to mouse receptors on muscle fibers and ECs and human receptors on tumor cells. The model can also be adapted to simulate mouse syngeneic tumors, where the tumor cells secrete VEGF_120_ and VEGF_164_; in this case, only mVEGF is present in the model. In this work, however, we have focused on human tumors. The molecular interactions between VEGF and its receptors are illustrated in Figure 8.

In addition to introducing the tumor compartment, we include VEGF interactions with two soluble factors: soluble VEGFR1 (sVEGFR1) and α2M and introduce VEGF secretion by ECs. Soluble VEGFR1 is secreted by ECs and transported throughout the body, enabling it to interact with VEGF in all compartments. The soluble factor α2M is present in two forms: native and active (α2M_fast_) (61). Both forms are present at high concentrations (nanomolar to micromolar levels) (62), and due to their size (720 kDa MW), we assume that both forms are confined to the blood compartment. The model predicts the levels of free VEGF in the tissue interstitium and in plasma. These soluble factors interfere with assays that measure VEGF concentration, making it difficult to distinguish between VEGF that is truly free versus VEGF that is bound to trapping molecules (63). Both sVEGFR1 and α2M can sequester VEGF and reduce the levels of free VEGF. Therefore, it is important to include these factors in the model.

We have also included VEGF secretion by ECs, as experimental studies demonstrate that EC are a source of VEGF (64, 65). The luminal and abluminal endothelial surfaces secrete VEGF, and luminal secretion is predicted to be a major determinant of plasma VEGF. Due to EC secretion of VEGF, the compartments are relatively autonomous, since the concentration of VEGF in each compartment is determined primarily by the secretion rate in that compartment, as well as the microenvironmental variables of the compartment; however, transport between compartments is also important.

**Figure 9:**
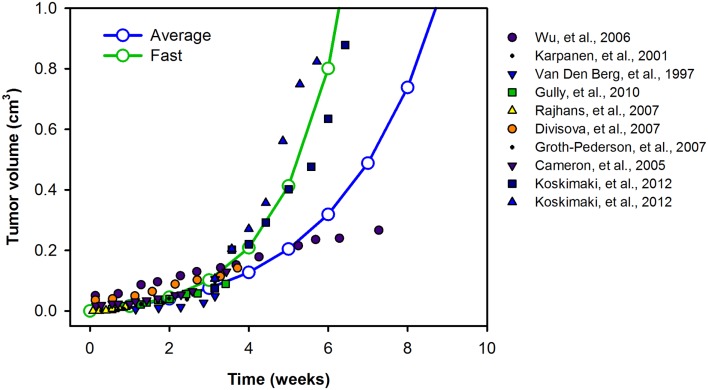
**Tumor growth profiles**. We investigate the growth profiles of two categories of tumors: average- (blue) and fast-growing (green) tumors, based on available experimental data. The data are fit to exponential curves, and the growth equations are given in the File 2 in Supplementary Material.

The model is described by 258 non-linear ordinary differential equations (ODEs), including 53 for the normal compartments, 126 for the blood, and 79 for the tumor compartment. In addition to the ODEs that describe how the species’ concentrations vary with time, we include an equation for the tumor volume, such that the model simulates VEGF distribution in tumor-bearing mice, immediately following inoculation of tumor cells. The initial tumor volume is 10^−6^ cm^3^. A sampling of experimental data for the volume of xenografts generated from MCF-7 and MDA-MB-231 breast cancer cells (66–74) reveals various growth profiles. We fit the data to exponential curves, accounting for a range of tumor growth profiles (Figure 9). The growth curves fit experimental data well, within the time scales used in the model (i.e., <6 weeks). In cases where the model is run for longer times, different growth curves should be used in order to capture the full range of tumor growth dynamics for the desired time scale. The complete set of equations, chemical reactions, and glossary of terms are given in File 2 in Supplementary Material.

### Simulation of administration of VEGF Trap

Experimental studies utilize a subcutaneous injection of VEGF Trap (“anti-VEGF”); however, the authors of the experimental study state that the bioavailability of the drug is the same whether injected subcutaneously or intravenously (12). The current model does not include a subcutaneous compartment; therefore, we simulate an intravenous injection, which inherently assumes that all of the drug appears in the blood. Injection lasts for 1 min (the duration does not affect the results, within limits) and is performed once the tumor reaches a particular volume, according to experimental methods described by Rudge et al. (12). Various doses of VEGF Trap are used, as reported by Rudge and coworkers (12) (0.5, 1, 2.5, 10, and 25 mg/kg).

### Sensitivity analysis

In order to understand the impact of various parameters, we perform variance-based global sensitivity analyses using the eFAST (75). The eFAST method estimates the sensitivity of model outputs (i.e., VEGF concentration) with respect to variations in model parameters. The three-compartment model is run multiple times with different parameter sets, where all parameters are varied from their baseline values. Variance for a parameter *i* is:

$$D_{i}=2\sum_{p=1}^{\infty }\left(A_{pj}^{2}+B_{pj}^{2}\right)$$

where *A_j_* and *B_j_* are the Fourier coefficients of the cosine series and sine series, respectively, for the frequency, *j*, associated with the parameter *i* and include harmonics, *p*, of the base frequency. The total variance in the output is:

$$D_{\text{total}}=2\sum_{j=1}^{\infty }\left(A_{j}^{2}+B_{j}^{2}\right)$$

The variances are used to estimate two indices that provide a measure the sensitivity: first-order FAST indices, *S_i_*, and the total FAST indices, *S*_T*i*_. The first-order indices measure the local sensitivity and do not account for interactions with other parameters:

$$S_{i}=\frac{D_{i}}{D_{\text{total}}}$$

The Total FAST indices measure of global sensitivity and take into account second- and higher-order interactions between parameters. *S*_T*i*_ are calculated by excluding the effects of the complementary set of other parameters:

$$S_{\text{T}i}=1-\frac{D_{\mathrm{ci}}}{D_{\text{total}}}$$

The eFAST method has been applied to systems biology models (76), and our laboratory has previous used the method to investigate the sensitivity of VEGFR2 signaling (77). In this work, we apply eFAST to investigate the sensitivity of steady state VEGF concentrations with respect to kinetic parameters, transport parameters, and receptor expression levels. We use Simlab 2.2 from Econometrics and Applied Statistics Unit EAS at the Joint Research Centre of the European Commission to implement eFAST.

### Numerical implementation

The model equations were implemented in MATLAB using the SimBiology toolbox and were solved with the Sundials solver. The model is available in SBML format at: http://www.jhu.edu/apopel/software.html

### Parameters

#### Geometry

The geometric parameters for the tumor compartment are summarized in Table A1 in Appendix. The tumor cells are assumed to have the same volume as the MCF-7 breast tumor cells, which have a mean diameter of 12 μm (78). A sphere of this diameter would have a volume and surface area of 905 μm^3^ and 452 μm^2^, respectively. However, since tumor cells are not spherical, we assume a dodecahedral cell of the same volume, which has a surface area of 497 μm^3^. The average luminal diameter of capillaries in growing MCF-7 xenografts is 13.94 μm (79), and imaging of tumor vasculature supports this value (80). We assume an EC thickness of 0.5 μm, which would yield a cylindrical cross-sectional area of 175 μm^2^ and an outer perimeter of 46.9 μm. However, microvessels are not cylindrical. Therefore, to find the true perimeter, we used a relationship between total perimeter and total cross-sectional area in breast cancer capillaries, where the increase in perimeter is 23% (81, 82), yielding a capillary perimeter of 57.7 μm.

The extracellular fluid volume fraction in the breast tumor xenografts has been shown to range from 33 to 76% (78). Another measurement reports the extracellular fluid volume in MCF-7 tumors to be 40% (83). We assume a value of 45%, which is divided into interstitial space and intravascular space. We set the volume fraction of intravascular space to be 10%, which is within the range of available experimental data (84–86). Given the capillary dimensions described above and an intravascular volume of 10%, the capillary density is calculated to be 655 capillaries/mm^2^. Based on a cell thickness of 0.5 μm, the volume occupied by the ECs of the microvessels is 1.5%. Cancer cells occupy the remaining tissue volume of 53.5%. The volume fractions of microvessels and tumor cells are then used to calculate the total surface area of all vessels and tumor cells per unit volume of tissue: 378 cm^2^ EC surface/cm^3^ tissue and 2939 cm^2^ tumor cell surface/cm^3^ tissue.

The interstitial space is composed of extracellular matrix (ECM), and basement membranes associated with the microvessels (endothelial basement membrane, EBM) and tumor cells (parenchymal basement membrane, PBM). The thickness of the basement membranes is assumed to be 50 and 30 nm, for the EBM and PBM, respectively, yielding volume fractions of 0.0081 and 0.0015 cm^3^/cm^3^ tissue. The remaining volume of the interstitial space is the ECM volume (34.04%).

Each region of the interstitial space can be represented as a porous medium that contains a solid fraction composed primarily of collagen that is unavailable to VEGF, and a fluid fraction that is accessible to VEGF. The size of the pores further limits the volume available for VEGF to diffuse. Therefore, the available volume in the ECM and basement membranes is calculated as the product of the volume, fluid fraction, and partition coefficient. The fluid fraction is the non-collagen fraction and is calculated by using the total collagen content in interstitial space. Given limited data for this measurement, we used 5%, the same value as in our previous models (24, 25, 87). The ratio of basement membrane collagen to total body collagen is assumed to be 0.3, which yields 0.0482 for the ratio of ECM collagen to total body collagen. The fluid fractions are then 0.7 for the basement membranes and 0.9318 for the ECM. The partition coefficient is the ratio of available fluid volume to interstitial fluid volume. We take 0.9 for the partition coefficient for the EBM (88), and the same value is used for the ECM and PBM, as it is difficult to distinguish basement membranes and the ECM (89). The available fluid volume for the ECM, EBM, and PBM are therefore 0.2916, 9.720 × 10^−4^, and 5.082 × 10^−3^ cm^3^/cm^3^ tissue, respectively.

#### Concentrations

Receptor densities and ECM binding site densities are listed in Table A2 in Appendix. VEGFR1, VEGFR2, and NRP1 on the luminal and abluminal surfaces of diseased EC surfaces and on tumor cells are based on quantitative flow cytometry measurements in ECs isolated from tumor tissue, as described in (25). We assume NRP2 surface concentration on tumor cells at the same level as NRP1.

#### Kinetics

To our knowledge, there are no data for the kinetics of mVEGF isoforms binding to glycosaminoglycan (GAG) chains or mouse receptors or cross-reactions between human and mouse isoforms and receptors. Therefore, we assume the kinetic rates for VEGF binding to and dissociation from receptors, co-receptors, and GAG chains in the ECM and basement membranes are the same as in our previous papers, based on experimental data (23–25, 87) and are given in Table A3 in Appendix. We use experimental data from Papadopoulos (11) for the on and off rates of VEGF binding to VEGF Trap.

#### Transport

Transport parameters for VEGF, anti-VEGF, and the VEGF/anti-VEGF complex are listed in Table A4 in Appendix. Parameters that govern transport between the normal and blood compartments are the same as in our previous model (23). Here, we explain specific transport parameters required for the addition of soluble factors sVEGFR1 and α2M and the tumor compartment. As in the previous model, myocytes are a source of VEGF and secrete the VEGF isoforms VEGF_120_ and VEGF_164_ at a ratio of 8:92 (90, 91). Additionally, tumor cells secrete VEGF into the tumor interstitium at a ratio of 50:50 for VEGF_121_:VEGF_165_, based on experimental quantification of mRNA isoform expression levels (92–96). Here, we also consider VEGF secretion by EC. We set the secretion ratio of VEGF_120_:VEGF_164_ by EC to be 10:90, similar to the isoform ratio in muscle tissue, since to our knowledge, this ratio has not been determined experimentally. Additionally, we assume normal and tumor EC secrete the same amount of VEGF; tumor EC are a small fraction of the total EC in the body, thus this assumption should not affect VEGF distribution. The rates of VEGF secretion by muscle fibers, EC, and tumor cells are determined by parameter optimization, fitting to experimental data from Rudge and coworkers (12).

This expanded model includes soluble factors sVEGFR1 and α2M. ECs are a source of sVEGFR1, and the rates of secretion by normal EC was set to 6 × 10^−3^ molecules/cell/s. Similar to VEGF secretion, we assume that sVEGFR1 secretion rate is the same for tumor EC. At steady state, the model estimated the distribution of sVEGFR1 in the body to be 0.4, 2.1, and 0.04 pM in the normal, blood, and tumor compartments, respectively. The level of sVEGFR1 in the plasma is within the range of experimental measurements, which range from 1 to 10 pM (97, 98). The clearance of α2M was set at 2.62 × 10^−3^ min^−1^, based on experimental measurements of the half-life, *t*_1/2_ (99), using ln(2)/*t*_1/2_. The synthesis of α2M was then estimated from mass balance at steady state, where the concentrations of native and active α2M are 1.4 μM (62) and 14 nM, respectively. We assume that the concentration of active α2M is 100-fold lower than that of the native form, based on experimental data for humans (100–102).

Molecular species are removed from the system via two mechanisms: plasma clearance and proteolytic degradation. The values of these parameters are in Table A4 in Appendix. For the normal endothelium, the permeability to sVEGFR1 and VEGF/sVEGFR1 is calculated using an empirical relation between the Stokes–Einstein radius, *a*_E_, and molecular weight [*a*_E_ = 0.483 × (MW)^0.386^], the corresponding theoretical macromolecular permeability-surface area product, PS (103), and the capillary surface area, *S*. Taking microvascular permeability as PS/*S*, and the calculated value is on the order of 10^−8^ cm/s, between the normal and blood compartments. Since tumor vasculature is more permeable than normal microvessels (104), we assume that the microvascular permeability between the tumor and blood is an order of magnitude higher than permeability between normal and blood for both VEGF and the anti-VEGF or complex. Therefore, the permeability to VEGF is 4 × 10^−7^ and 3 × 10^−7^ cm/s for the anti-VEGF and VEGF/anti-VEGF complex. The permeability to sVEGFR1 and VEGF bound to sVEGFR1 is 1.5 × 10^−7^ cm/s.

#### Parameter estimation

The estimation of the VEGF secretion by muscle fibers, ECs, and tumor cells was achieved using the “lsqnonlin” function in MATLAB, as previously described (23). This algorithm solves the non-linear least squares problem using the trust-region-reflective optimization algorithm (105, 106), minimizing the weighted sum of the squared residuals (WSSR):

$$\min WSSR\left(\theta \right)=\min\overset{n}{\underset{i=1}{\sum }}{\left[W_{i}\left(C_{\text{experimental},i}-C_{\text{simulation},i}\left(\theta \right)\right)\right]}^{2}$$

where *C*_experimental, *i*_ is the *i*th experimentally measured plasma concentration data point, *C*_simulation, *i*_(θ) is the *i*th simulated plasma concentration at the corresponding time point, *W_i_* is the weight taken to be 1/*C*_experimental, *i*_, and *n* is the total number of experimental measurements. The minimization is subject to the upper and lower bounds of the free parameters, θ.

The two-compartment model was used to determine the rate of VEGF secretion by muscle fibers and ECs (“normal” and “EC” secretion, respectively), clearance of free and bound VEGF Trap, dissociation constant of VEGF and VEGF Trap. These five free parameters were fit to experimental data for the concentration profiles of VEGF/VEGF Trap complex and unbound VEGF Trap in mice at different doses of VEGF Trap (12), with a total of 58 data points. The initial value of the secretion rates was generated within the lower and upper bounds of 1.5 × 10^−6^ and 2 molecules/cell/s, respectively. The lower bound corresponds to 10 pg/ml and was set based on the limit of detection of standard ELISA kits used to measure (63). The half-life of VEGF Trap in mouse serum has been reported as 72 h (107), which corresponds to a clearance rate of 1.6 × 10^−4^ min^−1^, assuming clearance rate is equal to ln(2)/half-life. The upper and lower bounds of the clearance rates were one order of magnitude above and below this value, respectively. The upper and lower bounds for the dissociation constant were set to 0.25 and 5 pM, based on experimental data (11, 12). The baseline value of permeability of the normal tissue to VEGF Trap is 3 × 10^−8^ cm/s, as described above, and the bounds were one order of magnitude above and below this value. The optimal parameter values are reported as the mean and standard deviation of the 20 runs.

We used the three-compartment model to determine the rate at which VEGF is secreted by tumor cells (“tumor secretion”) and permeability of diseased tissue to free and complexed VEGF Trap. Tumor secretion was optimized to fit experimental data for the systemic VEGF Trap levels (free and complexed) reported by Rudge et al. (12). Experimental data for two human tumor xenografts (A673 rhabdomyosarcoma and HT1080 fibrosarcoma) were used separately; the total number of data points was 11 for A673 tumors and 10 for HT1080 tumors. Twenty runs were performed for each tumor, which either followed the average (baseline) or fast growth profile. This yields two conditions for each tumor type. The optimal secretion rates are reported as the mean and standard deviation of the 20 runs and should be interpreted as a range of values, where the values are dependent on the tumor microenvironment, tumor type, and growth profile.

## Conflict of Interest Statement

The authors declare that the research was conducted in the absence of any commercial or financial relationships that could be construed as a potential conflict of interest.

## Supplementary Material

The Supplementary Material for this article can be found online at http://www.frontiersin.org/Molecular_and_Cellular_Oncology/10.3389/fonc.2013.00196/abstract

## Figures and Tables

**Figure A1:**
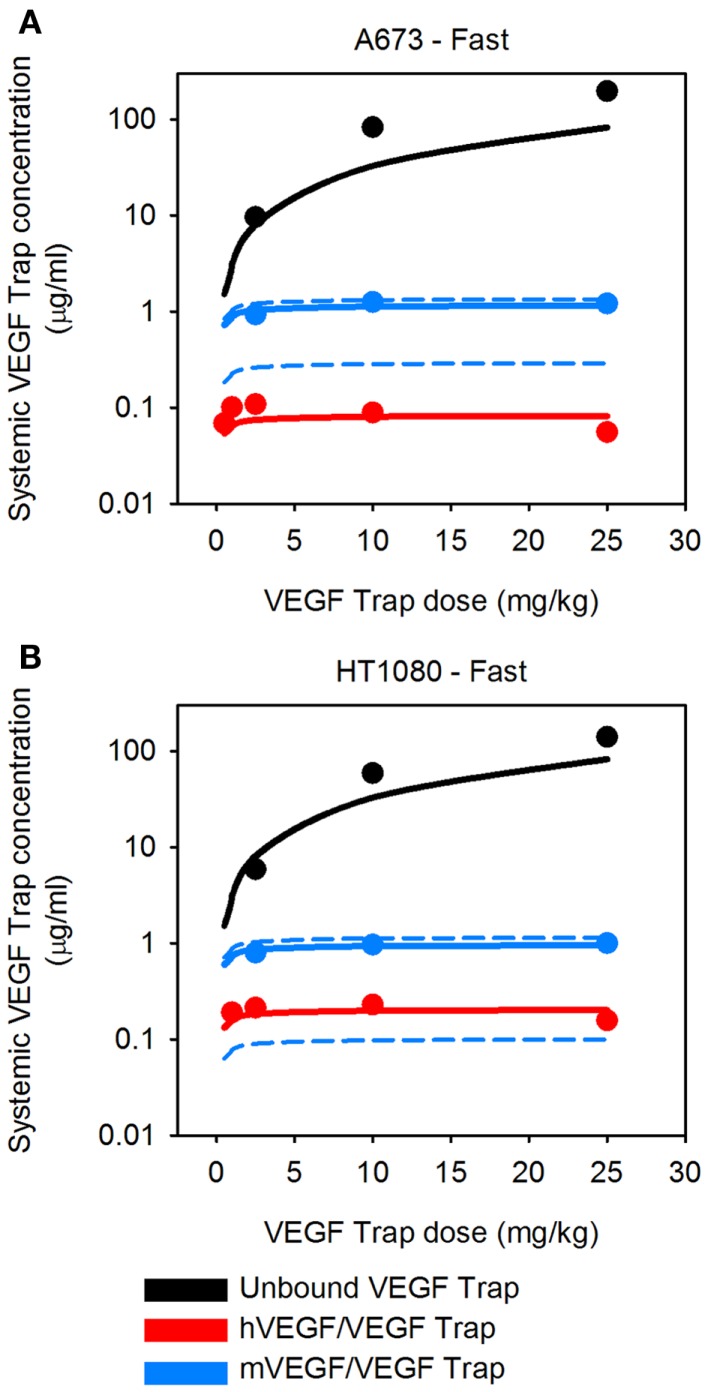
**Predicted systemic VEGF Trap levels for fast-growing tumors**. The model predicts the plasma levels of free VEGF Trap (black lines), mouse VEGF bound to VEGF Trap (blue lines), and human VEGF bound to VEGF Trap (red lines) for fast-growing tumors. VEGF Trap was administered twice per week for 2 weeks at doses of 0.5, 1, 2.5, 10, and 25 mg/kg. The simulated results are shown for the optimized model where the secretion rates of VEGF by myocytes, EC, and tumor cells were fit to experimental data (circles). We use the mean (solid lines) and 1 SD (dashed lines) of the fitted secretion rates. **(A)** A673 tumor; and **(B)** HT1080 tumor.

**Figure A2:**
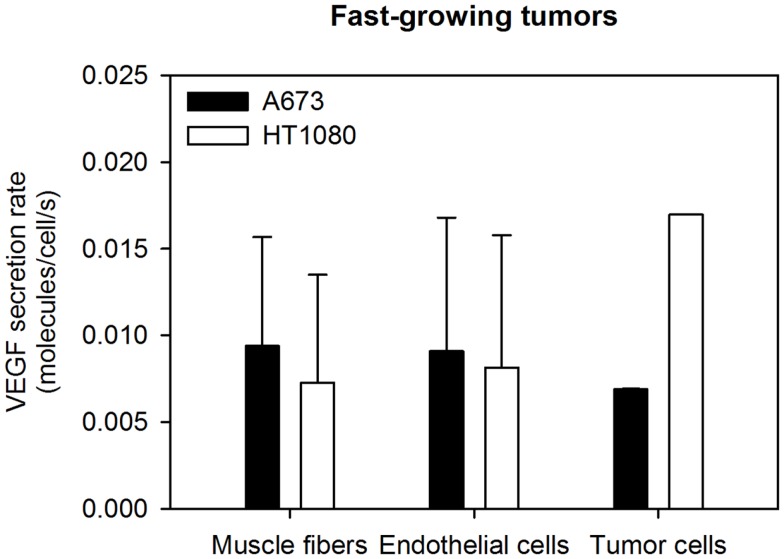
**Optimized VEGF secretion rates**. The model parameters were optimized to fit experimental data, and the values of normal, EC, and tumor VEGF secretion rates were determined. The mean optimal secretion rates and standard deviation of 20 optimization runs for fast-growing tumors are shown.

## Appendix

**Table A1:** **Geometric parameters**.

	Value	Units	Reference
**CANCER CELLS**			
Tumor cell external diameter	12	μm	Paran et al. (78)
Volume of one cell	905	μm^3^	Calculated (see manuscript)
Surface area of one cell	497	μm^2^	Calculated (see manuscript)
**MICROVESSELS**			
Average luminal diameter	13.9	μm	Schaefer et al. (79)
Endothelial cell thickness	0.5	μm	Based on normal microvessels (108)
Average external diameter	14.9	μm	Calculated (see manuscript)
Cross-sectional area of one vessel	175.3	μm^2^	Calculated (see manuscript)
Perimeter of one vessel	57.7	μm	Calculated (see manuscript)
Capillary density	655	Capillaries/mm^2^	Calculated (see manuscript)
**VOLUME FRACTIONS**			
Interstitial space	35.0%	cm^2^/cm^3^ tissue	Based on (78, 83)
Cancer cells	53.5%	cm^2^/cm^3^ tissue	Calculated (see manuscript)
Microvessels of which intravascular space	11.5%	cm^2^/cm^3^ tissue	Calculated (see manuscript)
	10.0%	cm^2^/cm^3^ tissue	Based on (84–86)
**SURFACE AREAS**			
Tumor cells	2939	cm^2^/cm^3^ tissue	Calculated (see manuscript)
Microvessels	378	cm^2^/cm^3^ tissue	Calculated (see manuscript)
**BASEMENT MEMBRANES (BM)**			
Thickness of tumor cell BM	30	nm	Based on (109)
Basement membrane volume (tumor cells) of which available to VEGF	0.00807	cm^3^/cm^3^	Calculated (see manuscript)
	0.00508	cm^3^/cm^3^ tissue	Calculated (see manuscript)
Thickness of microvessel BM	50	nm	Based on (109)
Basement membrane volume (microvessels) of which available to VEGF	0.00154	cm^3^/cm^3^ tissue	Calculated (see manuscript)
	0.000972	cm^3^/cm^3^ tissue	Calculated (see manuscript)
Extracellular matrix volume of which available to VEGF	0.3375	cm^3^/cm^3^ tissue	Calculated (see manuscript)
	0.2892	cm^3^/cm^3^ tissue	Calculated (see manuscript)

**Table A2:** **Concentrations in tumor compartment**.

	Value	Units
**VEGFR1**		
Luminal EC	3750	Dimers/EC
Abluminal EC	3750	Dimers/EC
Tumor	1100	Dimers/TC
**VEGFR2**		
Luminal EC	300	Dimers/EC
Abluminal EC	300	Dimers/EC
Tumor	550	Dimers/TC
**NRP1**		
Luminal EC	39,748	Dimers/EC
Abluminal EC	39,748	Dimers/EC
Tumor	39,500	Dimers/TC
**NRP2**		
Tumor	39,500	Dimers/TC
ECM binding density	0.75	μM
EBM binding density	13	μM
PBM binding density	13	μM

*EC, endothelial cell; TC, tumor cell*.

**Table A3:** **Kinetic parameters**.

	Value	Unit	Reference
**VEGF BINDING TO VEGFR1**			
*k*_on_	3 × 10^7^	M^−1^ s^−1^	Mac Gabhann and Popel (87), Stefanini et al. (24)
*k*_off_	10^−3^	s^−1^	Mac Gabhann and Popel (87), Stefanini et al. (24)
*K*_d_	33	pM	Mac Gabhann and Popel (87), Stefanini et al. (24)
**VEGF BINDING TO VEGFR2**			
*k*_on_	10^7^	M^−1^ s^−1^	Mac Gabhann and Popel (87), Stefanini et al. (24)
*k*_off_	10^−3^	s^−1^	Mac Gabhann and Popel (87), Stefanini et al. (24)
*K*_d_	100	pM	Mac Gabhann and Popel (87), Stefanini et al. (24)
**VEGF BINDING TO NRP1**			
*k*_on_	3.2 × 10^6^	M^−1^ s^−1^	Mac Gabhann and Popel (87), Stefanini et al. (24)
*k*_off_	10^−3^	s^−1^	Mac Gabhann and Popel (87), Stefanini et al. (24)
*K*_d_	312.5	pM	Mac Gabhann and Popel (87), Stefanini et al. (24)
**VEGF BINDING TO GAGs**			
*k*_on_	4.20 × 10^5^	M^−1^ s^−1^	Mac Gabhann and Popel (87), Stefanini et al. (24)
*k*_off_	10^−2^	s^−1^	Mac Gabhann and Popel (87), Stefanini et al. (24)
*K*_d_	24	pM	Mac Gabhann and Popel (87), Stefanini et al. (24)
**COUPLING OF NRP1 AND VEGFR1**			
*k*_c_	10^14^	(mol/cm^2^)^−1^ s^−1^	Mac Gabhann and Popel (87), Stefanini et al. (24)
*k*_off_	10^−2^	s^−1^	Mac Gabhann and Popel (87), Stefanini et al. (24)
**COUPLING OF NRP1 AND VEGFR2**			
*k*_cV165R2, N1_	3.1 × 10^13^	(mol/cm^2^)^−1^ s^−1^	Mac Gabhann and Popel (87), Stefanini et al. (24)
*k*_offV165R2, N1_	10^−3^	s^−1^	Mac Gabhann and Popel (87), Stefanini et al. (24)
*k*_cV165N1, R2_	10^14^	(mol/cm^2^)^−1^ s^−1^	Mac Gabhann and Popel (87), Stefanini et al. (24)
*k*_offV165N1, R2_	10^−3^	s^−1^	Mac Gabhann and Popel (87), Stefanini et al. (24)
**VEGFR INTERNALIZATION**			
*k*_int_	2.8 × 10^−4^	s^−1^	Mac Gabhann and Popel (87), Stefanini et al. (24)
**VEGF_121_ BINDING TO ANTI-VEGF**			
*k*_on_	3.75 × 10^8^	M^−1^ s^−1^	Calculated
*k*_off_	1.35 × 10^−5^	s^−1^	Papadopoulos et al. (11)
*K*_d_	0.36	pM	Papadopoulos et al. (11)
**VEGF_165_ BINDING TO ANTI-VEGF**			
*k*_on_	4.10 × 10^7^	M^−1^ s^−1^	Calculated
*k*_off_	2.01 × 10^−5^	s^−1^	Papadopoulos et al. (11)
*K*_d_	0.49	pM	Papadopoulos et al. (11)
**VEGF_120_ BINDING TO ANTI-VEGF**			
*k*_on_	2.15 × 10^7^	M^−1^ s^−1^	Calculated
*k*_off_	1.23 × 10^−5^	s^−1^	Papadopoulos et al. (11)
*K*_d_	0.572	pM	Papadopoulos et al. (11)
**VEGF_164_ BINDING TO ANTI-VEGF**			
*k*_on_	2.80 × 10^7^	M^−1^ s^−1^	Calculated
*k*_off_	1.64 × 10^−5^	s^−1^	Papadopoulos et al. (11)
*K*_d_	0.586	pM	Papadopoulos et al. (11)
**VEGF BINDING TO α2M**			
*k*_on_	25	M^−1^ s^−1^	Calculated
*k*_off_	10^−4^	s^−1^	Assumed
*K*_d_	4.0	μM	Bhattacharjee et al. (110)
**VEGF BINDING TO α2M_FAST_**			
*k*_on_	2.4 × 10^2^	M^−1^ s^−1^	Calculated
*k*_off_	10^−4^	s^−1^	Assumed
*K*_d_	0.42	μM	Bhattacharjee et al. (110)
**sVEGFR1 BINDING TO VEGF**			
*k*_on_	3 × 10^7^	M^−1^ s^−1^	Assumed, based on VEGF binding to VEGFR1
*k*_off_	10^−3^	s^−1^	Assumed
*K*_d_	33	pM	Assumed
**sVEGFR1 BINDING TO NRP1**			
*k*_on_	5.6 × 10^6^	M^−1^ s^−1^	Calculated
*k*_off_	10^−2^	s^−1^	Assumed, based on VEGFR1 coupling to NRP1
*K*_d_	1.8	nM	Fuh et al. (111)
**sVEGFR1 BINDING TO GAGs**			
*k*_on_	4.20 × 10^5^	M^−1^ s^−1^	Assumed, based on VEGF_165_ binding to GAG
*k*_off_	10^−2^	s^−1^	Assumed
*K*_d_	24	pM	Assumed

**Table A4:** **Transport parameters**.

	Value	Unit	Reference
**PERMEABILITY BETWEEN NORMAL AND BLOOD**			
VEGF	4.0 × 10^−8^	cm/s	Stefanini et al. (24)
Anti-VEGF and VEGF/anti-VEGF complex	3.0 × 10^−8^	cm/s	Stefanini et al. (24)
Soluble VEGFR1	1.5 × 10^−8^	cm/s	Calculated, see text
Soluble VEGFR1/VEGF complex	1.5 × 10^−8^	cm/s	Calculated, see text
**PERMEABILITY BETWEEN TUMOR AND BLOOD**			
VEGF	4.0 × 10^−7^	cm/s	Assumed, see text
Anti-VEGF and VEGF/anti-VEGF complex	3.0 × 10^−7^	cm/s	Assumed, see text
Soluble VEGFR1	1.5 × 10^−7^	cm/s	Assumed, see text
Soluble VEGFR1/VEGF complex	1.5 × 10^−7^	cm/s	Assumed, see text
**CLEARANCE**			
VEGF	2.3 × 10^−1^	min^−1^	Folkman (112)
Anti-VEGF	8.9 × 10^−4^	min^−1^	Yen et al. (23)
VEGF/anti-VEGF complex	2.8 × 10^−4^	min^−1^	Yen et al. (23)
Soluble VEGFR1	3.0 × 10^−4^	min^−1^	Wu et al. (113)
Soluble VEGFR1/VEGF complex	3.0 × 10^−4^	min^−1^	Wu et al. (113)
Alpha-2-macroglobulin (α2M)	2.6 × 10^−3^	min^−1^	Hudson et al. (99)
α2M/VEGF complex	2.6 × 10^−3^	min^−1^	Assumed, based on α2M
α2M/VEGF/anti-VEGF complex	2.6 × 10^−3^	min^−1^	Assumed, based on α2M
Activated alpha-2-macroglobulin (α2M_fast_)	2.4 × 10^−1^	min^−1^	Imber and Pizzo (114)
α2M/VEGF complex	2.6 × 10^−3^	min^−1^	Assumed, based on α2M_fast_
**DEGRADATION**			
Soluble VEGFR1	1.2 × 10^−2^	min^−1^	Assumed based on VEGF
Soluble VEGFR1/VEGF complex	1.2 × 10^−2^	min^−1^	Assumed based on VEGF
**SYNTHESIS**			
Alpha-2-macroglobulin	1.8 × 10^10^	Molecules/cm^3^ tissue/s	Calculated, see text
Activated alpha-2-macroglobulin	1.6 × 10^10^	Molecules/cm^3^ tissue/s	Calculated, see text

## References

[1] Rennel ES Harper SJ Bates DO Therapeutic potential of manipulating VEGF splice isoforms in oncology. Future Oncol (2009) 5:703–12 10.2217/fon.09.33 19519209 PMC2879319

[2] Dokun AO Annex BH The VEGF165b “ICE-o-form” puts a chill on the VEGF story. Circ Res (2011) 109:246–7 10.1161/CIRCRESAHA.111.249953 21778432 PMC3196354

[3] Koch S Tugues S Li X Gualandi L Claesson-Welsh L Signal transduction by vascular endothelial growth factor receptors. Biochem J (2011) 437:169–83 10.1042/BJ20110301 21711246

[4] Olsson A-K Dimberg A Kreuger J Claesson-Welsh L VEGF receptor signalling – in control of vascular function. Nat Rev Mol Cell Biol (2006) 7:359–71 10.1038/nrm1911 16633338

[5] Cao Y Therapeutic angiogenesis for ischemic disorders: what is missing for clinical benefits? Discov Med (2010) 9:179–84 20350482

[6] Roy RS Roy B Sengupta S Emerging technologies for enabling proangiogenic therapy. Nanotechnology (2011) 22:494004 10.1088/0957-4484/22/49/494004 22101316

[7] Al-Latayfeh M Silva PS Aiello LP Antiangiogenic therapy for ischemic retinopathies. Cold Spring Harb Perspect Med (2012) 2:a006411 10.1101/cshperspect.a006411 22675660 PMC3367538

[8] Jayson GC Hicklin DJ Ellis LM Antiangiogenic therapy – evolving view based on clinical trial results. Nat Rev Clin Oncol (2012) 9:297–303 10.1038/nrclinonc.2012.8 22330688

[9] Genentech, Inc Avastin Prescribing Information. (2011) [accessed September 2011]. Available from: http://www.avastin.com/avastin/hcp/overview/about/dosing/index.html

[10] Gaya A Tse V A preclinical and clinical review of aflibercept for the management of cancer. Cancer Treat Rev (2012) 38:484–93 10.1016/j.ctrv.2011.12.008 22264850

[11] Papadopoulos N Martin JD Ruan Q Rafique A Rosconi MP Shi E Binding and neutralization of vascular endothelial growth factor (VEGF) and related ligands by VEGF Trap, ranibizumab and bevacizumab. Angiogenesis (2012) 15:171–85 10.1007/s10456-011-9249-6 22302382 PMC3338918

[12] Rudge JS Holash J Hylton D Russell M Jiang S Leidich R VEGF Trap complex formation measures production rates of VEGF, providing a biomarker for predicting efficacious angiogenic blockade. Proc Natl Acad Sci U S A (2007) 104:18363–70 10.1073/pnas.0708865104 18000042 PMC2141784

[13] Singh M Ferrara N Modeling and predicting clinical efficacy for drugs targeting the tumor milieu. Nat Biotechnol (2012) 30:648–57 10.1038/nbt.2286 22781694

[14] Jain RK Normalizing tumor microenvironment to treat cancer: bench to bedside to biomarkers. J Clin Oncol (2013) 31:2205–18 10.1200/JCO.2012.46.3653 23669226 PMC3731977

[15] Peirce SM Computational and mathematical modeling of angiogenesis. Microcirculation (2008) 15:739–51 10.1080/10739680802220331 18720228 PMC3125711

[16] Qutub AA Mac Gabhann F Karagiannis ED Vempati P Popel AS Multiscale models of angiogenesis. IEEE Eng Med Biol Mag (2009) 28:14–31 10.1109/MEMB.2009.931791 19349248 PMC3077679

[17] Stefanini MO Qutub AA Mac Gabhann F Popel AS Computational models of VEGF-associated angiogenic processes in cancer. Math Med Biol (2012) 29:85–94 10.1093/imammb/dqq025 21266494 PMC4104688

[18] Byrne HM Dissecting cancer through mathematics: from the cell to the animal model. Nat Rev Cancer (2010) 10:221–30 10.1038/nrc2808 20179714

[19] Young RJ Reed MWR Anti-angiogenic therapy: concept to clinic. Microcirculation (2012) 19:115–25 10.1111/j.1549-8719.2011.00147.x 22078005

[20] Duda DG Munn LL Jain RK Can we identify predictive biomarkers for antiangiogenic therapy of cancer using mathematical models? J Natl Cancer Inst (2013) 105:762–5 10.1093/jnci/djt114 23670727 PMC3672078

[21] Wehland M Bauer J Magnussion NE Infanger M Grimm D Biomarkers for anti-angiogenic therapy in cancer. Int J Mol Sci (2013) 14:9338–64 10.3390/ijms14059338 23629668 PMC3676786

[22] Finley SD Popel AS Effect of tumor microenvironment on tumor VEGF during anti-VEGF treatment: systems biology predictions. J Natl Cancer Inst (2013) 105:802–11 10.1093/jnci/djt093 23670728 PMC3672077

[23] Yen P Finley SD Engel-Stefanini MO Popel AS A two-compartment model of VEGF distribution in the mouse. PLoS ONE (2011) 6:e27514 10.1371/journal.pone.0027514 22087332 PMC3210788

[24] Stefanini MO Wu FTH Mac Gabhann F Popel AS A compartment model of VEGF distribution in blood, healthy and diseased tissues. BMC Syst Biol (2008) 2:77 10.1186/1752-0509-2-77 18713470 PMC2562372

[25] Finley SD Engel-Stefanini MO Imoukhuede PI Popel AS Pharmacokinetics and pharmacodynamics of VEGF-neutralizing antibodies. BMC Syst Biol (2011) 5:193 10.1186/1752-0509-5-193 22104283 PMC3229549

[26] Finley SD Popel AS Predicting the effects of anti-angiogenic agents targeting specific VEGF isoforms. AAPS J (2012) 14:500–9 10.1208/s12248-012-9363-4 22547351 PMC3385824

[27] Chauhan VP Stylianopoulos T Martin JD Popovic Z Chen O Kamoun WS Normalization of tumour blood vessels improves the delivery of nanomedicines in a size-dependent manner. Nat Nanotechnol (2012) 7:383–8 10.1038/nnano.2012.45 22484912 PMC3370066

[28] Gavin TP Ruster RS Carrithers JA Zwetsloot KA Kraus RM Evans CA No difference in the skeletal muscle angiogenic response to aerobic exercise training between young and aged men. J Physiol (2007) 585:231–9 10.1113/jphysiol.2007.143198 17884919 PMC2375453

[29] Hellsten Y Rufener N Nielsen JJ Hoier B Krustrup P Bangsbo J Passive leg movement enhances interstitial VEGF protein, endothelial cell proliferation, and eNOS mRNA content in human skeletal muscle. Am J Physiol Regul Integr Comp Physiol (2008) 294:R975–82 10.1152/ajpregu.00677.2007 18094062

[30] Hansen-Algenstaedt N Nielsen JJ Saltin B Hellsten Y Exercise training normalizes skeletal muscle vascular endothelial growth factor levels in patients with essential hypertension. J Hypertens (2010) 28:1176–85 10.1097/HJH.0b013e3283379120 20179634

[31] Hoier B Rufener N Bojsen-Moller J Bangsbo J Hellsten Y The effect of passive movement training on angiogenic factors and capillary growth in human skeletal muscle. J Physiol (2010) 588:3833–45 10.1113/jphysiol.2010.190439 20693292 PMC2998230

[32] Hoier B Nordsborg N Andersen S Jensen L Nybo L Bangsbo J Pro- and anti-angiogenic factors in human skeletal muscle in response to acute exercise and training. J Physiol (2012) 590:595–606 10.1113/jphysiol.2011.216135 22155930 PMC3379703

[33] Hoier B Passos M Bangsbo J Hellsten Y Intense intermittent exercise provides weak stimulus for VEGF secretion and capillary growth in skeletal muscle. Exp Physiol (2013) 98:585–97 10.1113/expphysiol.2012.067967 22962287

[34] Le X-F Mao W Lu C Thornton A Heymach JV Sood AK Specific blockade of VEGF and HER2 pathways results in greater growth inhibition of breast cancer xenografts that overexpress HER2. Cell Cycle (2008) 7:3747–58 10.4161/cc.7.23.7212 19029832 PMC2757147

[35] Bjorndahl M Cao R Eriksson A Cao Y Blockage of VEGF-induced angiogenesis by preventing VEGF secretion. Circ Res (2004) 94:1443–50 10.1161/01.RES.0000129194.61747.bf 15192038

[36] Kavitha CV Agarwal C Argawal R Deep G Asiatic acid inhibits pro-angiogenic effects of VEGF and human gliomas in endothelial cell culture models. PLoS ONE (2011) 6:e22745 10.1371/journal.pone.0022745 21826202 PMC3149605

[37] van der Bilt ARM van Scheltinga AGTT Timmer-Bosscha H Schroder CP Pot L Kosterink JGW Measurement of tumor VEGF-A levels with 89Zr-bevacizumab PET as an early biomarker for the antiangiogenic effect of everolimus treatment in an ovarian cancer xenograft model. Clin Cancer Res (2012) 18:6306–14 10.1158/1078-0432.CCR-12-0406 23014526

[38] Leith JT Michelson S Secretion rates and levels of vascular endothelial growth factor in clone A or HCT-8 human colon tumour cells as a function of oxygen concentration. Cell Prolif (1995) 28:415–30 10.1111/j.1365-2184.1995.tb00082.x 7548442

[39] Rofstad EK Halsor EF Vascular endothelial growth factor, interleukin 9, platelet-derived endothelial cell growth factor, and basic fibroblast growth factor promote angiogenesis and metastasis in human melanoma xenografts. Cancer Res (2000) 60:4932–8 10987309

[40] Pilch H Schlenger K Steiner E Brockerhoff P Knapstein P Vaupel P Hypoxia-stimulated expression of angiogenic growth factors in cervical cancer cells and cervical cancer-derived fibroblasts. Int J Gynecol Cancer (2001) 11:137–42 10.1046/j.1525-1438.2001.011002137.x 11328412

[41] Salnikov AV Heldin N-E Stuhr LB Wiig H Gerber H-P Reed RK Inhibition of carcinoma cell-derived VEGF reduces inflammatory characteristics in xenograft carcinoma. Int J Cancer (2006) 119:2795–802 10.1002/ijc.22217 17019708

[42] Sugimoto H Hamano Y Charytan D Cosgrove D Kieran M Sudhakar A Neutralization of circulating vascular endothelial growth factor (VEGF) by anti-VEGF antibodies and soluble VEGF receptor 1 (sFlt-1) induces proteinuria. J Biol Chem (2003) 278:12605–8 10.1074/jbc.C300012200 12538598

[43] Bocci G Man S Green SK Francia G Ebos JM du Manoir JM Increased plasma vascular endothelial growth factor (VEGF) as a surrogate marker for optimal therapeutic dosing of VEGF receptor-2 monoclonal antibodies. Cancer Res (2004) 64:6616–25 10.1158/0008-5472.CAN-04-0401 15374976

[44] Keyes KA Mann L Cox K Treadway P Iversen P Chen Y-F Circulating angiogenic growth factor levels in mice bearing human tumors using Luminex Multiplex technology. Cancer Chemother Pharmacol (2003) 51:321–7 12721760 10.1007/s00280-003-0572-5

[45] Rullman E Rundqvist H Wagsater D Fischer H Eriksson P Sundberg CJ A single bout of exercise activates matrix metalloproteinase in human skeletal muscle. J Appl Physiol (2007) 102:2346–51 10.1152/japplphysiol.00822.2006 17255365

[46] Kut C Mac Gabhann F Popel AS Where is VEGF in the body? A meta-analysis of VEGF distribution in cancer. Br J Cancer (2007) 97:978–85 10.1038/sj.bjc.6603923 17912242 PMC2360423

[47] Bernaards C Hegde P Chen D Holmgren E Zheng M Jubb AM Circulating vascular endothelial growth factor (VEGF) as a biomarker for bevacizumab-based therapy in metastatic colorectal, non-small cell lung, and renal cell cancers: analysis of phase III studies. J Clin Oncol (2010) 28:10519

[48] Sanmartin E Jantus E Blasco A Sirera R Caballero C Gallach S Plasma levels of VEGF-A and VEGFR-2 in advanced NSCLC. J Clin Oncol (2010) 28:10623

[49] Willett CG Duda DG di Tomaso E Boucher Y Ancukiewicz M Sahani DV Efficacy, safety, and biomarkers of neoadjuvant bevacizumab, radiation therapy, and fluorouracil in rectal cancer: a multidisciplinary phase II study. J Clin Oncol (2009) 27:3020–6 10.1200/JCO.2008.21.1771 19470921 PMC2702234

[50] Hoff BA Bhojani MS Rudge J Chenevert TL Meyer CR Galban S DCE and DW-MRI monitoring of vascular disruption following VEGF-Trap treatment of a rat glioma model. NMR Biomed (2011) 25:935–42 10.1002/nbm.1814 22190279 PMC4307830

[51] Zhang Q-X Magover CJ Mack CA Budenbender KT Ko W Rosengart T Vascular endothelial growth factor is the major angiogenic factor in omentum: mechanism of the omentum-mediated angiogenesis. J Surg Res (1997) 67:147–54 10.1006/jsre.1996.4983 9073561

[52] Yuan A Lin C-Y Chou C-H Shih C-M Chen C-Y Cheng H-W Functional and structural characteristics of tumor angiogenesis in lung cancers overexpressing different VEGF isoforms assessed by DCE- and SSCE-MRI. PLoS ONE (2011) 6:e16062 10.1371/journal.pone.0016062 21283766 PMC3024413

[53] Woolard J Wang W-Y Bevan HS Qiu Y Morbidelli L Pritchard-Jones RO VEGF165b, an inhibitory vascular endothelial growth factor splice variant: mechanism of action, in vivo effect on angiogenesis and endogenous protein expression. Cancer Res (2004) 64:7822–35 10.1158/0008-5472.CAN-04-0934 15520188

[54] Bates DO Catalano PJ Symonds KE Varey AHR Ramani P O’Dwyer PJ Association between VEGF splice isoforms and progression-free survival in metastatic colorectal cancer patients treated with bevacizumab. Clin Cancer Res (2012) 18:6384–91 10.1158/1078-0432.CCR-12-2223 23104894 PMC3602975

[55] Achen MG Stacker SA Vascular endothelial growth factor-D: signaling mechanisms, biology, and clinical relevance. Growth Factors (2012) 30:283–96 10.3109/08977194.2012.704917 22817635

[56] Benedito R Rocha SF Woeste M Zamykal M Radtke F Casanovas O Notch-dependent VEGFR3 upregulation allows angiogenesis without VEGF-VEGFR2 signalling. Nature (2012) 484:110–4 10.1038/nature10908 22426001

[57] Chen JC Chang YW Hong CC Yu YH Su JL The role of the VEGF-C/VEGFRs axis in tumor progression and therapy. Int J Mol Med (2012) 14:88–107 10.3390/ijms14010088 23344023 PMC3565253

[58] Battinelli EM Markens BA Italiano JE Jr. Release of angiogenesis regulatory proteins from platelet alpha granules: modulation of physiologic and pathologic angiogenesis. Blood (2011) 118:1359–69 10.1182/blood-2011-02-334524 21680800 PMC3152500

[59] Yankeelov TE Lepage M Chakravarthy A Broome EE Niermann KJ Kelley MC Integration of quantitative DCE-MRI and ADC mapping to monitor treatment response in human breast cancer. Magn Reson Imaging (2007) 25:1–13 10.1016/j.mri.2006.09.006 17222711 PMC2634832

[60] Mitchell EP Targeted therapy for metastatic colorectal cancer: role of aflibercept. Clin Colorectal Cancer (2012) 12(2):73–85 10.1016/j.clcc.2012.08.001 23102896

[61] Barrett AJ Starkey PM The interaction of alpha 2-macroglobulin with proteinases. Characteristics and specificity of the reaction, and a hypothesis concerning its molecular mechanism. Biochem J (1973) 133:709–24 4201304 10.1042/bj1330709 PMC1177761

[62] Webb DJ Wen J Lysiak JJ Umans L Van Leuven F Gonias SL Murine a-macroglobulins demonstrate divergent activities as neutralizers of transforming growth factor-b and as inducers of nitric oxide synthesis. J Biol Chem (1996) 271:24982–8 10.1074/jbc.271.40.24982 8798779

[63] Jelkmann W Pitfalls in the measurement of circulating vascular endothelial growth factor. Clin Chem (2001) 47:617–23 11274009

[64] Lee S Chen TT Barber CL Jordan MC Murdock J Desai S Autocrine VEGF signaling is required for vascular homeostasis. Cell (2007) 130:691–703 10.1016/j.cell.2007.06.054 17719546 PMC3010851

[65] dela Paz NG Walshe TE Leach L Saint-Geniez M D’Amore PA Role of shear-stress-induced VEGF expression in endothelial cell survival. J Cell Sci (2012) 125:831–43 10.1242/jcs.084301 22399811 PMC3311927

[66] Van den Berg CL Cox GN Stroh CA Hilsenbeck SG Weng C-N McDermott MJ Polyethylene glycol conjugated insulin-like growth factor binding protein 1 (IGFBP-1) inhibits growth of breast cancer in athymic mice. Eur J Cancer (1997) 33:1108–13 10.1016/S0959-8049(97)00071-3 9376191

[67] Karpanen T Egeblad M Karkkainen MJ Kubo H Yla-Herttuala S Jaattela M Vascular endothelial growth factor C promotes tumor lymphangiogenesis and intralymphatic tumor growth. Cancer Res (2001) 61:1786–90 11280723

[68] Cameron IL Sun L-Z Short N Hardman WE Williams CD Therapeutic Electromagnetic Field (TEMF) and gamma irradiation on human breast cancer xenograft growth, angiogenesis and metastasis. Cancer Cell Int (2005) 5:23 10.1186/1475-2867-5-17 16045802 PMC1190196

[69] Divisova J Kuiatse I Lazard Z Weiss H Vreeland F Hadsell DL The growth hormone receptor antagonist pegvisomant blocks both mammary gland development and MCF-7 breast cancer xenograft growth. Breast Cancer Res Treat (2006) 98(3):315–27 10.1007/s10549-006-9168-1 16541323

[70] Wu Y Hooper AT Zhong Z Witte L Bohlen P Rafii S The vascular endothelial growth factor receptor (VEGFR-1) supports growth and survival of human breast carcinoma. Int J Cancer (2006) 119:1519–29 10.1002/ijc.21865 16671089

[71] Groth-Pedersen L Ostenfeld MS Hoyer-Hansen M Nylandsted J Jaattela M Vincristine induces dramatic lysosomal changes and sensitizes cancer cells to lysosome-destabilizing siramesine. Cancer Res (2007) 67:2217–25 10.1158/0008-5472.CAN-06-3520 17332352

[72] Rajhans R Nair S Holden AH Kumar R Takmel RR Vadlamudi RK Oncogenic potential of the nuclear receptor coregulator proline-, glutamic acid-, leucine-rich protein 1/modulator of the nongenomic actions of the estrogen receptor. Cancer Res (2007) 67:5505–12 10.1158/0008-5472.CAN-06-3647 17545633 PMC2774841

[73] Gully CP Zhang F Chen J Yeung JA Valazquez-Torres G Wang E Antineoplastic effects of an Aurora B kinase inhibitor in breast cancer. Mol Cancer (2010) 9:42 10.1186/1476-4598-9-42 20175926 PMC2839967

[74] Koskimaki JE Rosca EV Rivera CG Lee E Chen W Pandey NB Serpin-derived peptides are antiangiogenic and suppress breast tumor xenograft growth. Trxansl Oncol (2012) 5:92–7 22496925 10.1593/tlo.11244 PMC3323930

[75] Saltelli A Bolado R An alternative way to compute Fourier amplitude sensitivity test (FAST). Comput Stat Data Anal (2008) 26:445–60 10.1016/S0167-9473(97)00043-1

[76] Marino S Hogue IB Ray CJ Kirschner DE A methodology for performing global uncertainty and sensitivity analysis in systems biology. J Theor Biol (2008) 254:178–96 10.1016/j.jtbi.2008.04.011 18572196 PMC2570191

[77] Tan WH Popel AS Mac Gabhann F Computational model of Gab1/2-dependent VEGFR2 pathway to Akt activation. PLoS ONE (2013) 8:e67438 10.1371/journal.pone.0067438 23805312 PMC3689841

[78] Paran Y Bendel P Margalit R Degani H Water diffusion in the different microenvironments of breast cancer. NMR Biomed (2004) 17:170–80 10.1002/nbm.882 15229930

[79] Schaefer C Schroeder M Fuhrhop I Viezens L Otten J Fiedler W Primary tumor dependent inhibition of tumor growth, angiogenesis, and perfusion of secondary breast cancer in bone. J Orth Res (2011) 29:1251–8 10.1002/jor.21402 21381098

[80] Kim E Stamatelos SK Cebulla J Bhujwalla ZM Popel AS Pathak AP Multiscale imaging and computational modeling of blood flow in the tumor vasculature. Ann Biomed Eng (2012) 40:2425–41 10.1007/s10439-012-0585-5 22565817 PMC3809908

[81] Olewniczak S Chosia M Kwas A Kram A Domagala W Angiogenesis and some prognostic parameters of invasive ductal breast carcinoma in women. Pol J Pathol (2002) 53:183–8 12597334

[82] Olewniczak S Chosia M Kolodziej B Kwas A Kram A Domagala W Angiogenesis as determined by computerised image analysis and the risk of early relapse in women with invasive ductal breast carcinoma. Pol J Pathol (2003) 54:53–9 12817881

[83] Hassid Y Furman-Haran E Margalit R Eilam R Degani H Noninvasive magnetic resonance imaging of transport and interstitial fluid pressure in ectopic human lung tumors. Cancer Res (2006) 66:4159–66 10.1158/0008-5472.CAN-05-3289 16618737

[84] Lewin M Bredow S Sergeyev N Marecos E Bodganov A Jr. Weissleder R In vivo assessment of vascular endothelial growth factor-induced angiogenesis. Int J Cancer (1999) 83:798–802 10.1002/(SICI)1097-0215(19991210)83:6<798:AID-IJC16>3.0.CO;2-W 10597197

[85] Bogin L Margalit R Mispelter J Degani H Parametric imaging of tumor perfusion using flow- and permeability-limited tracers. J Magn Reson Imaging (2002) 16:289–99 10.1002/jmri.10159 12205585

[86] Cao M Liang Y Shen C Miller KD Stantz KM Developing DCE-CT to quantify intra-tumor heterogeneity in breast tumors with different angiogenic phenotype. IEEE Trans Med Imaging (2009) 28:861–71 10.1109/TMI.2008.2012035 19150783

[87] Mac Gabhann F Popel AS Targeting neuropilin-1 to inhibit VEGF signaling in cancer: comparison of therapeutic approaches. PLoS Comp Biol (2006) 2:e180 10.1371/journal.pcbi.0020180 17196035 PMC1761657

[88] Yuan F Krol A Tong S Available space and extracellular transport of macromolecules: effects of pore size and connectedness. Ann Biomed Eng (2001) 29:1150–8 10.1114/1.1424915 11853267

[89] Hashizume H Baluk P Morikawa S McLean JW Thurston G Roberge S Openings between defective endothelial cells explain tumor vessel leakiness. Am J Pathol (2000) 156:1363–80 10.1016/S0002-9440(10)65006-7 10751361 PMC1876882

[90] Ng YS Rohan R Sunday ME Demello DE D’Amore PA Differential expression of VEGF isoforms in mouse during development and in the adult. Dev Dyn (2001) 220:112–21 10.1002/1097-0177(2000)9999:9999<::AID-DVDY1093>3.0.CO;2-D 11169844

[91] Gustafsson T Ameln H Fischer H Sundberg CJ Timmons JA Jansson E VEGF-A splice variants and related receptor expression in human skeletal muscle following submaximal exercise. J Appl Physiol (2005) 98:2137–46 10.1152/japplphysiol.01402.2004 15661835

[92] Cheung N Wong MP Yuen ST Leung SY Chung LP Tissue-specific expression pattern of vascular endothelial growth factor isoforms in the malignant transformation of lung and colon. Hum Pathol (1998) 29:910–4 10.1016/S0046-8177(98)90195-2 9744306

[93] Yuan A Yu CJ Luh KT Lin FY Kuo SH Yang PC Quantification of VEGF mRNA expression in non-small cell lung cancer using a real-time quantitative reverse transcription-PCR assay and a comparison with quantitative competitive reverse transcription-PCR. Lab Invest (2000) 2000:11 11092527 10.1038/labinvest.3780177

[94] Stimpfl M Tong D Fasching B Schuster E Obermair A Leodolter S Vascular endothelial growth factor splice variants and their prognostic value in breast and ovarian cancer. Clin Cancer Res (2002) 8:2253–9 12114428

[95] Ljungberg B Jacobsen J Haggstrom-Rudolfssson S Rasmuson T Lindh G Grankvist K Tumor vascular endothelial growth factor (VEGF) mRNA in relation to serum VEGF protein levels and tumour progression in human renal cell carcinoma. Urol Res (2003) 31:335–40 10.1007/s00240-003-0346-x 14574539

[96] Zygalaki E Tsaroucha EG Kaklamanis L Lianidou ES Quantitative real-time reverse transcription-PCR study of the expression of vascular endothelial growth factor (VEGF) splice variants and VEGF receptors (VEGFR-1 and VEGFR-2) in non-small cell lung cancer. Clin Chem (2007) 53:1433–9 10.1373/clinchem.2007.086819 17599955

[97] Roland CL Dineen SP Lynn KD Sullivan LA Dellinger MT Sadegh L Inhibition of vascular endothelial growth factor reduces angiogenesis and modulates immune cell infiltration of orthotopic breast cancer xenografts. Mol Cancer Ther (2009) 8:1761–71 10.1158/1535-7163.MCT-09-0280 19567820

[98] Blankenberg FG Levashova Z Sarkar SK Pizzonia J Backer MV Bacher JM Noninvasive assessment of tumor VEGF receptors in response to treatment with pazopanib: a molecular imaging study. Transl Oncol (2010) 3:56–64 20165696 10.1593/tlo.09271 PMC2822454

[99] Hudson NW Kehoe JM Koo PH Mouse alpha-macroglobulin. Structure, function, and a molecular model. Biochem J (1987) 248:837–45 2449173 10.1042/bj2480837 PMC1148625

[100] Banks RE Evans SW Van Leuven F Alexander D McMahon MJ Whicher JT Measurement of the “fast” or complexed form of α2-macroglobulin in biological fluids using a sandwich enzyme immunoassay. J Immunol Methods (1990) 126:13–20 10.1016/0022-1759(90)90006-H 1689358

[101] Zucker S Lysik RM Zarrabi MH Fiore JJ Strickland DK Proteinase-alpha2 macroglobulin complexes are not increased in plasma of patients with cancer. Int J Cancer (1991) 48:399–403 10.1002/ijc.2910480316 1710207

[102] Jespersen MH Jensen J Rasmussen LH Ejlersen E Moller-Petersen J Sperling-Petersen HU The reference range for complexed a2-macroglobulin human plasma: development of a new enzyme linked in immunosorbent assay (ELISA) for quantitation of complexed a2-macroglobulin. Scand J Clin Lab Invest (1993) 53:639–48 10.1080/00365519309092565 7505478

[103] Garlick DG Renkin EM Transport of large molecules from plasma to interstitial fluid and lymph in dogs. Am J Physiol (1970) 219:1595–605 5485678 10.1152/ajplegacy.1970.219.6.1595

[104] Goel S Duda DG Xu L Munn LL Boucher Y Fukumura D Normalization of the vasculature for treatment of cancer and other diseases. Physiol Rev (2011) 91:1071–121 10.1152/physrev.00038.2010 21742796 PMC3258432

[105] Coleman TF Li Y On the convergence of reflective Newton methods for large-scale nonlinear minimization subject to bounds. Math Program (1994) 67:189–224 10.1007/BF01582221

[106] Coleman TF Li Y An interior, trust region approach for nonlinear minimization subject to bounds. SIAM J Optim (1996) 6:418–45 10.1137/0806023

[107] Wachsberger PR Burd R Cardi C Thakur M Daskalakis C Holash J VEGF trap in combination with radiotherapy improves tumor control in u87 glioblastoma. Int J Radiat Oncol Biol Phys (2007) 67:1526–37 10.1016/j.ijrobp.2006.11.011 17234361

[108] Hall JE The circulation. In: Hall JE editor. Guyton and Hall Textbook of Medical Physiology. 12th ed. Philadelphia, PA: W.B. Saunders Company (2011). p. 177–90

[109] Baluk P Morikawa S Haskell A Mancuso M McDonald DM Abnormalities of basement membrane on blood vessels and endothelial sprouts in tumors. Am J Pathol (2003) 163:1801–15 10.1016/S0002-9440(10)63540-7 14578181 PMC1892429

[110] Bhattacharjee G Asplin IR Wu SM Gawdi G Pizzo SV The conformation-dependent interaction of alpha-2-macroglobulin with vascular endothelial growth factor. J Biol Chem (2000) 275:26806–11 10862607 10.1074/jbc.M000156200

[111] Fuh G Garcia KC de Vos AM The interaction of neuropilin-1 with vascular endothelial growth factor and its receptor flt-1. J Biol Chem (2000) 275:26690–5 10842181 10.1074/jbc.M003955200

[112] Folkman J Angiogenesis in cancer, vascular rheumatoid and other disease. Nat Med (1995) 1:27–31 10.1038/nm0195-27 7584949

[113] Wu FTH Stefanini MO Mac Gabhann F Popel AS A compartment model of VEGF distribution in humans in the presence of soluble VEGF receptor-1 acting as a ligand trap. PLoS ONE (2009) 4:e5108 10.1371/journal.pone.0005108 19352513 PMC2663039

[114] Imber MJ Pizzo SV Clearance and binding of two electrophoretic “fast” forms of human alpha-2-macroglobulin. J Biol Chem (1981) 256:8134–9 6167573

